# Mechanisms of Protein Sequence Divergence and Incompatibility

**DOI:** 10.1371/journal.pgen.1003665

**Published:** 2013-07-25

**Authors:** Alon Wellner, Maria Raitses Gurevich, Dan S. Tawfik

**Affiliations:** Department of Biological Chemistry, Weizmann Institute of Science, Rehovot, Israel; Brown University, United States of America

## Abstract

Alignments of orthologous protein sequences convey a complex picture. Some positions are utterly conserved whilst others have diverged to variable degrees. Amongst the latter, many are non-exchangeable between extant sequences. How do functionally critical and highly conserved residues diverge? Why and how did these exchanges become incompatible within contemporary sequences? Our model is phosphoglycerate kinase (PGK), where lysine 219 is an essential active-site residue completely conserved throughout Eukaryota and Bacteria, and serine is found only in archaeal PGKs. Contemporary sequences tested exhibited complete loss of function upon exchanges at 219. However, a directed evolution experiment revealed that two mutations were sufficient for human PGK to become functional with serine at position 219. These two mutations made position 219 permissive not only for serine and lysine, but also to a range of other amino acids seen in archaeal PGKs. The identified trajectories that enabled exchanges at 219 show marked sign epistasis - a relatively small loss of function with respect to one amino acid (lysine) versus a large gain with another (serine, and other amino acids). Our findings support the view that, as theoretically described, the trajectories underlining the divergence of critical positions are dominated by sign epistatic interactions. Such trajectories are an outcome of rare mutational combinations. Nonetheless, as suggested by the laboratory enabled K219S exchange, given enough time and variability in selection levels, even utterly conserved and functionally essential residues may change.

## Author Summary

Orthologs are proteins in different species sharing the same function and structure. However, the mechanisms that underline the divergence of different sequences from a single ancestor remain unclear, particularly because many amino acid exchanges between orthologs result in loss of function (incompatibility). We aimed at disentangling an ancient divergence event within the active-site of a universally spread enzyme that mediates ATP synthesis. Using laboratory evolution experiments, we found that an exchange in a functionally critical active-site residue that is incompatible within contemporary orthologs is enabled by few mutations. These mutations lead to transition sequences in which, unlike the extant sequences, a wide range of amino acids is tolerated. Our experiment reveals the properties of these transition sequences that may resemble the historical ancestral states that underlined this divergence event, and the mechanisms that led to incompatibility within the contemporary orthologs. Our results support theoretical predictions and reshape our understanding of protein structure-function. That a given position is entirely conserved and essential for function does not indicate that it will never exchange, but rather, that the exchange may depend on changes in many other positions.

## Introduction

Universally-spread proteins suggest a common ancestor dating back to the last universal common ancestor (LUCA), ∼3.5 billion years ago [1]–[3]. They indicate that most positions have drifted from their original state, but many, typically active-site positions, remained unchanged. However, many [4], if not most [5], exchanges between orthologous sequences are incompatible — namely, amino acids that are functional in one sequence can be deleterious in related ones. Thus, as protein sequences evolve, they navigate a rugged fitness landscape formed by a complex network of epistatic interactions [4], [6]–[11]. Computational analysis suggests that this ruggedness distinctly relates to sign epistasis, and not to any other mechanism that could limit divergence (e.g. a large fraction of mutations that are intolerable) [12]. Sign epistasis relates to a qualitative rather than a quantitative effect, namely a mutation that is beneficial within one genetic background but deleterious at another. This form of epistasis results in a very slow yet constant divergence rate, suggesting that protein sequences have not yet reached the limits of their divergence potential [6]. Experimentally, protein sequence divergence and epistasis have been studied [4], [10], [13], [14]. It remains unclear, however, how sequence space is traversed at crucial active-site positions, particularly in positions that have a key functional role and are highly conserved. Similarly, the mechanism(s) leading to incompatibility — i.e., an originally tolerated exchange becoming unacceptable in orthologous sequences, are also unclear.

Our case study regards an ancient substitution that occurred within the active site of the highly ubiquitous phosphoglycerate kinase (PGK). PGK is an essential glycolytic enzyme that catalyzes the interconversion of 1,3-bisphosphoglycerate and ADP to 3-phosphoglycerate and ATP. Its enzymatic activity is directly related to growth and thereby to organismal fitness [15]. PGK can be traced back to LUCA [1], [2], [16] and its evolutionary rate is extremely slow. It is categorized in the 10^th^ percentile of slowly evolving proteins [17]. Indeed, the sequence identity between the human and the archaeon *Methanococcus mazei* PGKs is 32%, and nearly all active-site positions are entirely conserved in all known PGKs. However, Position 219 comprises an exception. It is lysine in all known bacterial and eukaryotic PGKs, and this lysine was shown to play a critical role in ATP binding and phosphate transfer [18]. The ancestor of all PGKs is also predicted to have had a lysine at position 219. Archaeal PGKs, however, possesses mostly serine or threonine at this position, but never a lysine.

Although a transition from lysine to serine and subsequently to other amino acids had occurred at an early point in PGK's history, this event was singular, as no successive exchanges seem to have occurred within the lysine clade. Indeed, as described below, the K219S exchange renders human and *Escherichia coli* PGKs non-functional, and the reciprocal S219K replacement in the archaeal *M. mazei* PGK has the same effect. We aimed at disentangling the incompatibility at PGK's position 219 and thus address the following conundrum. On the one hand, exchanges at numerous other positions erased the trails that had historically enabled to traverse sequence space at this position - the closest archaeal sequences (the serine/threonine clade) and bacterial/eukaryotic sequences (the lysine clade) are separated by 250 amino acid exchanges. Further, the traversing trails are presumed to be intricate and scarce [6]. On the other hand, it is assumed that “functional proteins must form a continuous network which can be traversed by unit mutational steps without passing through nonfunctional intermediates” [19]. Unraveling this network is fundamental to our understanding of molecular evolution.

## Results

### Sequence divergence at PGK's active-site

**Figure 1:**
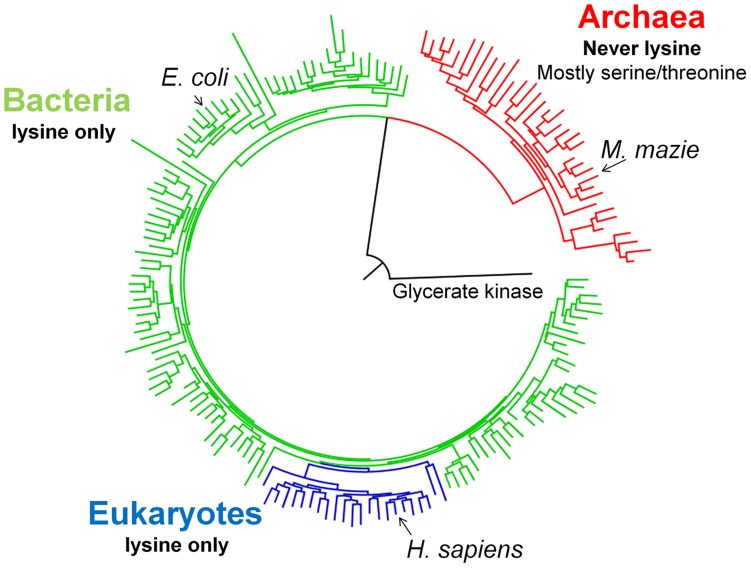
A schematic PGK phylogenetic tree. Shown are 175 PGK protein sequences rooted with Glycerate kinase from *Neisseria meningitides* (Detailed analysis, a complete tree (620 sequences) and alignment are given as Supplementary information). All bacterial sequences (in green) and eukaryotic sequences (blue) have lysine at position 219. None of the archeal sequences (in red) harbors a lysine at 219, with the most abundant amino acids being serine (45/73) and threonine (12/73). Redundancy threshold was increased to 93% to create a minimal set of sequences.

As previously shown [20], the phylogenetic tree of 620 diverse PGK sequences largely follows the species tree with a clear separation between the three kingdoms of life (Figure 1). Overall, within kingdoms, conservation is high. For instance, the amino acid identity of human relative to chicken PGK is ∼90%, and ∼60% relative to yeast (*Saccharomyces cerevisiae*). However, between kingdoms, the average sequence identity drops to approximately 35%. The sequence identity between human and *E. coli* PGKs, for example, is 38%. The archaeal kingdom is the most diverged one with, on average, ∼30% identity to bacterial or eukaryotic PGKs. Nonetheless, the active-site residues known to play a role in PGK's enzymatic function are conserved throughout, with only two exceptions: positions 219 and 336 (numbering is according to human PGK; Figure S1). All bacteria and eukaryotes have lysine at position 219, as does the inferred ancestor of all PGKs (lysine predicted with 81% posterior probability; data not shown). In contrast, no archaeal PGK has a lysine at position 219, with serine being most abundant (Figure 1). The phylogenetic pattern of position 336 is less consistent. Nonetheless, exchanges in both 219 and 336 are incompatible (see below). Given its clear phylogenetic pattern, and the well-defined catalytic role of lysine 219 in human PGK, we focused on the exchange at position 219. We chose human PGK (thereafter marked as hPGK), rather than that of *E. coli*, because of the large available body of biochemical and structural data [18], [21]–[24]. Specifically, lysine 219 of hPGK was shown to be directly involved in substrate binding and domain closure upon catalysis [18]. Lysine 219 also contributes to formation of the active site transition state electrostatics [21] (Figure S2).

### Construction and characterization of *E. coli-*Δ*pgk*

An *in-vivo* selection system for PGK was developed by inactivating *E.coli*'s *pgk* gene. An *E. coli*-Δ*pgk* strain is publicly available. This strain was obtained via transposon mutagenesis and screening for loss of ability to grow on glucose as the sole carbon source [25]. However, although this strain exhibited no PGK activity, our sequencing of the *pgk* open reading frame, and of 200 bps of its flanking regions, revealed no mutations. Previous attempts to obtain a *pgk* chromosomal deletion in *E. coli* had failed [26]. We could, however, knock out the *pgk* gene when the transformed cells were plated on minimal growth media supplemented only with glycerol and succinate. Indeed, a bidirectional pathway such as glycolysis can be circumvented by supplying the metabolites from both ends of the pathway [25]. The resultant Δ*pgk* strain was not only unable to utilize glucose as a carbon source (as the previously reported Δ*pgk* strain [25]), but its growth on glycerol-succinate was completely inhibited when glucose was added at any level beyond 1 µM. The previously reported *E. coli*-Δ*pgk* therefore confers only a partial phenotype, and the previous failure to obtain a *pgk* knockout [26] may relate to plating on rich medium.

### Incompatibility of serine-lysine exchanges

**Figure 2:**
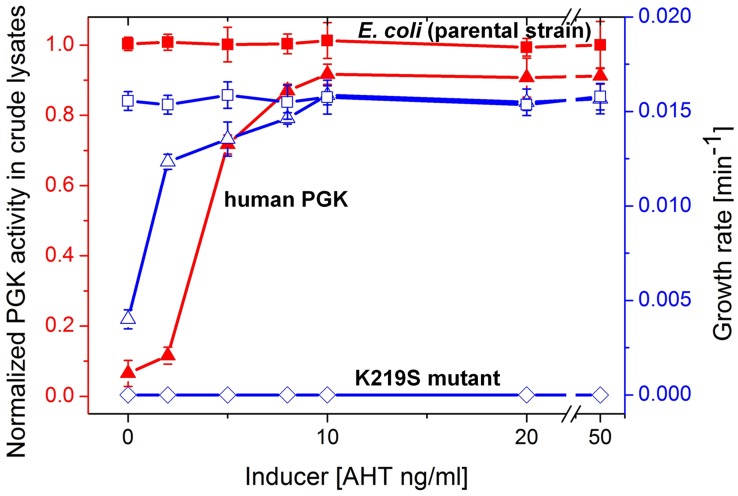
Growth rates and PGK cell lysate activities. Plotted are growth rates (open symbols, right Y axis) and levels of PGK enzymatic activity in crude cell lysates (closed symbols, left Y axis) as a function of the expression inducer (AHT) concentration. Shown are the parental *E. coli* strain (squares) and its *pgk* knockout (Δ*pgk*). The latter was supplemented with the pZA plasmid encoding wild-type hPGK (lysine 219; triangles), or its K219S mutant (diamonds). Cells were grown in glycerol-succinate media supplemented with 5 mM glucose and increasing AHT concentrations. Cell suspensions were normalized to the same cell density, lysed, and PGK activity levels in the lysates were determined. The lysate activities were normalized to the activity of the parental *E. coli* strain MG1655 (3±0.34×10^5^ µmole product/min/mg dry cells). Growth rates are presented as the inverse of the shortest doubling time among time intervals [46] (the shortest doubling time was observed with *E. coli* MG1655, and was 63±2.8 mins). Error bars represent the standard deviation of ≥3 independent measurements (growth, lysis, and assays).

The human *pgk* gene was placed within a plasmid dubbed pZA-hPGK under the tight regulation of a tetracycline-inducible promoter. At ≥8 ng/ml of the inducer AHT (anhydrous tetracycline), *E. coli*-Δ*pgk* cells harboring this plasmid (∼40 copies per cell [27]) grew at the same rate as the parental strain with the endogenous, chromosomal *E. coli* PGK in glycerol-succinate media with 5 mM glucose. At the range of 0–8 ng/ml AHT, cellular PGK activity levels and growth rates were concomitantly dependent on the inducer's concentration (Figure 2).

When lysine 219 of the plasmid-encoded hPGK was mutated to serine, no growth on glucose containing media was observed (Figure 2). In agreement, a 400-fold decrease in *k_cat_/K_M_* value was measured for the K219S mutant (Table 1, Figure S3). The K219S exchange in *E.coli*'s PGK resulted in a similar effect, namely no growth on media containing ≥1 µM glucose with the mutant expressed from the pZA plasmid (data not shown). The reciprocal replacement, S219K, had a similar effect in the archaeal, *M. mazei* PGK. Expressed from the pZA plasmid, wild-type *M. mazei* PGK supported growth on glucose similar to *E. coli* and human PGK. However, its S219K mutant showed no growth in glucose containing media (data not shown).

**Table 1:** Representative variants underlining the directed evolution of hPGK- serine 219.

Position ↓	Clone →	Wild type	G1-v5	G2-v15	G4-v4	G6-v9	G7-v12
Asn35							Ser (10%)
Asp67					His		
Ile116							Phe
Met233							Val (14%)
Met239					**Ile** (75%)	**Ile** (52%)	**Ile** (90%)
Thr254				Ala (12.5%)	Ala (42%)		
Phe257						Tyr (29%)	Tyr (50%)
Ala262			Val^a^				
Thr287				Ile			
Arg349				Ser			Ser (37%)
Lys352					Glu		
Ser363							Gly (30%)
Thr384						Ala (14%)	Ala (20%)
Ala397						Val (35%)	
Glu403			**Asp** (33%)	Gly (50%)	**Asp** (67%)		**Asp** (90%)
Growth rate (min)	Ser 219	ND	134.5±2.5	77.4±1.9	73.4±3.7	75±3.4	63.8±1.0
	Lys 219	63.8±1.8	62.94±1.6	66.3±2	79.5±2.24	101.4±3.7	101±3.3
*k_cat_/K_M_* [mM^−1^ sec^−1^]	Ser 219	1.55±0.46	9.6±1.5	27.8±4.5	60±3.3	171±7.3	62.5±3.7
	Lys 219	687±5^a^	38.4±6	141.2±7.2	39.9±6.3	60.9±3.4	35.8±6.4

Noted are sequence changes, growth rates and *k_cat_/K_M_* values of representative variants along the laboratory trajectory. Variants annotation: G stands for the round number from which a variant was isolated, and v denotes the variant's sequential number within this round. The left column denotes the sequence composition and rate parameters of wild-type hPGK. In bold are mutations that appeared at ≥90% of the variants by the last round (near complete fixation). Mutations that were not fixed appear in standard letters and their percentage of occurrence is denoted in brackets (no percentage is indicated for mutations that appeared only once in >100 variants).

a Note that *k_cat_/K_M_* for wild type hPGK we measured was ∼11-fold lower than previously reported [18]. The origins of this difference are unknown, yet the rates for various mutants were measured by us under the very same conditions as wild-type, thus providing consistency within the reported set. The ± mark represents the S.D within 3 independent measurements.

### Directed evolution of hPGK-serine 219

We sought to identify mutations that could rescue the enzymatic function of hPGK-serine 219 (second-site suppressor mutations). To this end, seven iterative rounds of random mutagenesis and selection were performed to gradually increase the ability of K219S mutated hPGK genes to confer growth on glucose. The gene library was generated by error-prone PCR of the hPGK-serine 219 gene, giving rise to an average of 2.5 mutations per gene. To minimize the chances of serine 219 being reverted to lysine, the serine codon at position 219 was designed to be TCT, whereby all three bases must mutate simultaneously to give lysine. Nonetheless, mutations to lysine and wild-type contaminants still dominated the first and second rounds (appearing in 10 to 20 percent of the surviving clones). Thus, during all rounds, a special cloning protocol was applied to prevent exchanges of the serine at position 219 (see Text S1).

Because hPGK-serine 219 gave no growth on glucose when expressed from the pZA plasmid, we began by over-expressing it from the high copy plasmid pZUC (∼900 copies per cell [28]), thus enabling some growth on glucose supplemented plates (∼1 mm colonies after 72 hours). The selection pressure was gradually strengthened by decreasing the inducer concentration (thus lowering the expression level), decreasing incubation time, and subsequently, by re-cloning the libraries to plasmids of lower copy numbers (Methods, Tables 2 and S1). In each round, 10–20 randomly chosen surviving colonies were isolated and the harbored hPGK genes were sequenced. All surviving colonies were subsequently pooled. The plasmid DNA was isolated and the *pgk* gene was subjected to further mutagenesis, re-cloning and selection.

**Table 2:** Summary of experimental conditions for the seven rounds of directed evolution.

Round #	Plasmid	Mutagenesis strategy	Average number of Mutations per gene	Inducer AHT ng/ml	Time of incubation	Total # of transformants^3^	# of transformants on selection plates
1	pZUC	Mutazyme^1^	2.5	0	24	10^5^	10^3^
2	pZE	Gene shuffling^2^ and TAQ polymerase	ND	100	48	5×10^3^	4.5×10^2^
3	pZE	TAQ polymerase	≤1	50	24	6×10^3^	5×10^2^
4	pZE	TAQ polymerase	≤1	10	24	10^4^	6×10^2^
5	pZE	TAQ polymerase	≤1	8	18	10^4^	3×10^2^
6	pZA	TAQ polymerase	≤1	8	18	10^4^	10^3^
7	pZA	Mutazyme	2.5	5	18	7×10^3^	3×10^2^

1 Random mutagenesis was preformed with either an error-prone polymerase (Mutazyme), or with an ordinary TAQ polymerase that lacks a proof-reading domain. In all cases, 60 cycles of PCR were conducted as part of the protocol for preventing mutations at position 219 (see Text S1). Mutational rates were calculated by implementing the same mutagenesis conditions on wild type *pgk* and sequencing of 10 randomly picked clones. The application of Mutazyme resulted in average of 2.5 mutations per gene, whereas 60 cycles with the ordinary TAQ polymerase incorporated, on average, about one mutation per gene. In rounds 1 and 7, Mutazyme was used in the first PCR, and TAQ polymerase was used in the second. In rounds 2 to 7, TAQ polymerase was used for both PCR reactions.

2 In the second round, an *in vitro* homologous recombination protocol was applied (DNA shuffling). Overall, 90 cycles of PCR with TAQ polymerase were conducted since the shuffling protocol includes an additional PCR (for a detailed protocol, see supplemental methods).

3 The total number of transformants was determined by plating a fraction of the transformation on glycerol-succinate plates (where no selection for PGK activity occurs) with kanamycin (the Δ*pgk* selection marker) and ampicillin (the plasmid selection marker). The number of viable transformants on selection plates relates to colonies that appeared on the glucose-contacting selection plates.

By round 5, the sequences of randomly sampled variants indicated near complete convergence with 4–7 substitutions within and around hPGK's active site (the mean being 5.2±1.1 substitutions per gene; the sequence composition of the evolved variants is discussed below). Indeed, no further improvement in growth, or substantial sequence changes, could be observed in the subsequent round. In the seventh round, with the aim of isolating the most active hPGK K219S variants, we conducted random mutagenesis by error-prone PCR with the selected gene pool of round 6 as template, followed by 3 cycles of selection on glucose plates (extracting the plasmid pool, retransforming and plating). Eight of the largest colonies of round 7 were examined. They exhibited growth rates identical to wild-type hPGK, but similar catalytic efficiencies (*k_cat_/K_M_* values; Table 1 and S2) relative to the best variants from round 6. The likely explanation for having improved growth yet identical catalytic efficiency is that surface mutations from basic to acidic residues (lysine to glutamate) were highly abundant in round 7 variants (Figure S4). These led to a reduction in PGK's pI value, thus meeting the conditions of the *E. coli* cytoplasm (the pI of hPGK is 8.75 [29] whereas the pH of the *E. coli* cytoplasm is 7.5 [30]). As such, these surface mutations are possibly resulting in improved stability and elevated amounts of soluble protein [31], therefore increasing enzyme dose rather than specifically compensating for the serine-lysine exchange. Such compensatory mutations that increase stability and/or solubility and therefore enzyme doses are commonly observed, e.g. in the clinical emergence of drug resistance [32].

### Identifying serine-lysine transition sequences

**Figure 3:**
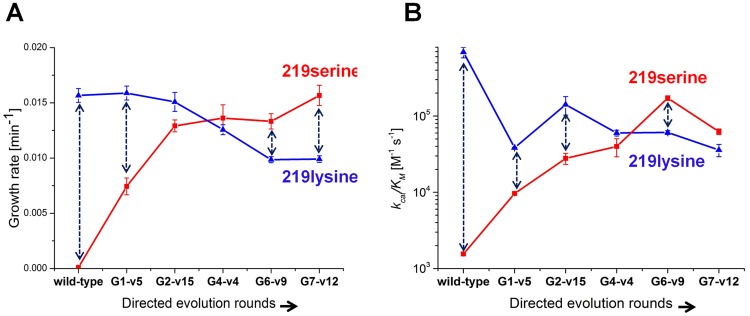
Growth rates and catalytic efficiencies of the evolved variants. (a) Growth rates of the evolved serine 219 hPGK variants and of their lysine 219 counterparts. (b) *k_cat_/K_M_* values of the same variants (log scale). Error bars represent S.D of 8 measurements. Each double headed dashed arrow connects a 219 serine variant with its 219 lysine counterpart.

Variants that represented the sequence trends in each round along the described evolutionary trajectory were characterized (the choice of variants is described in Methods). Since these variants were originally selected in various copy number plasmids, they were all re-cloned to the low copy number pZA plasmid and re-sequenced to confirm their mutational compositions. Cell cultures (Δ*pgk*) harboring the evolved hPGKs were grown in glycerol-succinate media supplemented with 5 mM glucose and 8 ng/ml AHT, such that the growth rates were most sensitive to the enzymatic efficiency of these variants relative to wild-type hPGK (Figure 2). The evolved variants exhibited increasingly higher growth rates that matched the order of their appearance along the trajectory (Figure 3a).

Next, serine 219 was exchanged back to lysine by site-directed mutagenesis of the evolved hPGK variants. The series of evolved hPGK variants exhibited an opposite trend to the one observed with the original serine 219 variants - growth rates gradually decreased with sequential rounds of evolution. The loss of activity with lysine at position 219, however, was mild relative to the gain of activity with serine (Figure 3a). Thus, a zone of relative neutrality with respect to position 219 has been revealed in hPGK's sequence space. These sequences could theoretically facilitate serine-lysine transitions. For example, variants G2-v15 and G4-v4 (G stands for the round number from which the variant was isolated, and v is the variant's sequential number within this round) can accommodate either lysine or serine at position 219 yet exhibit growth rates that are not very far from wild-type. The growth rates of these transition variants in media containing only glucose were lower than in medium containing glucose plus succinate and glycerol (Figure S5). Nonetheless, growth was clearly observed in these variants, with either lysine or serine at 219. In contrast, the K219S mutant of hPGK conferred no growth in any glucose containing media, even when succinate and glycerol comprised the primary carbon source (Figure 3a).

To validate the observed trend in growth rates, these representative variants were purified and assayed *in vitro*. The *k_cat_/K_M_* values and growth rates were generally correlated (Figure 3b). Most variations related to lower *k_cat_/K_M_* values relative to growth rates (e.g. variant G7-v12), as discussed above, are attributed to surface mutations.

### Sequence composition of the serine-lysine transition variants

Two mutations appeared in nearly all the library selected variants from round 4 to round 7: methionine at position 239 exchanged to isoleucine, and glutamate at position 403 to aspartate (Table 1). Indicatively, these mutations appear in nearly all the transition variants - i.e., variants in which both serine and lysine are accommodated with little effect on growth rates (Table S2). Although located away from the active site and ∼9 Å from lysine 219, glutamate 403 plays a role in hPGK's catalytic cycle, and specifically in the inter-domain motions that relate to substrate binding and phosphoryl transfer [21]. Phylogenetically, positions 219 and 403 seem correlated: >98% of sequences that have a lysine at position 219 (535/547) have glutamate at position 403. None of the 72 sequences in the archaeal clade (non-lysine 219) have glutamate at position 403, with alanine being the most frequent amino acid (Figure S6a).

Methionine 239 is in close proximity to lysine 219 (3.5 Å). However, phylogenetically, it is weakly correlated to position 403. Amongst the sequences that have a lysine at position 219, methionine is most frequent (76%; as in human PGK), and the remaining sequences have mostly isoleucine or leucine at position 239 (117 and 14, respectively). Amongst archaeal PGKs, none have a methionine whilst most sequences have a valine (60/73) and the remaining have isoleucine or leucine (Figure S6b).

Sequence wise, the outlier was variant G6-v9 that also exhibited the highest *k_cat_/K_M_* value. Instead of the E403D mutation present in all other transition sequences, it contained the A397V mutation that appeared in 35% of the selected variants of this round (Table 1). However, A397V was purged by the 7^th^ round that was completely dominated by the aforementioned pair of mutations (M239I *plus* E403D). Indeed, E403D and A397V never appeared together, suggesting they might have a beneficial effect individually but interact with negative epistasis (see below).

### The transition sequences are highly permissive at position 219

**Figure 4:**
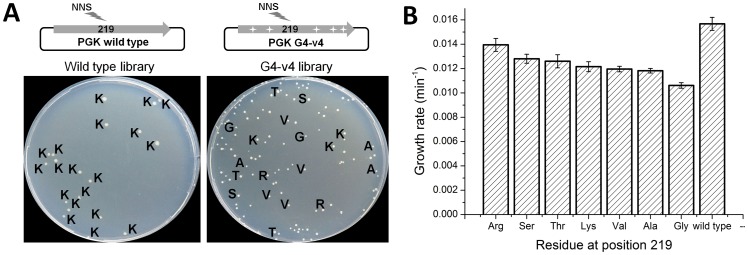
Evolved variant G4-v4 is highly permissive to exchanges at position 219. (a) Libraries encoding all 20 possible amino acids at position 219 were generated for wild-type hPGK, and it's evolved variant G4-v4. Aprox. 500 transformants from each library were plated on glucose selection plates. Only 4% of the wild-type library clones were viable, all carrying lysine at 219 (amino acids are denoted with single letter codes). In contrast, 26% of the G4-v4 library transformants were viable. These were variable in colony size and growth rates and carried a range of amino acids. With the exception of glycine (smallest colonies and lowest growth rate), the amino acids tolerated by variant G4-v4 are all observed within archaeal PGKs. Conversely, leucine is the only amino acid observed in archaeal PGKs (rarely though, 5/72) but not in G4-v4's viable clones. (b) The growth rates of the G4-v4 variants carrying different amino acids at position 219. The values comprise are average of 12 parallel measurements. Also shown is the growth rate for wild type hPGK for comparison.

Archaeal PGKs have not only diverged away from the absolute conservation of lysine at position 219 seen along the bacterial and eukaryote clades, but have also drifted considerably at this position. Archaeal PGKs with serine, but also with threonine, valine, arginine and leucine are found within relatively close phylogenetic proximity (Figure S7). Since the laboratory-evolved transition sequences accepted serine, and were also functional with lysine, we tested whether they might accept additional amino acids at position 219. Thus, a variable NNS codon (N, any nucleotide; S, G or C), that can give rise to any one of the 20 possible amino acids (but only one stop codon) was introduced at position 219, of both the evolved transition variant G4-v4 and wild-type hPGK. The resulting gene libraries were cloned into the pZA plasmid, transformed into Δ*pgk* cells and selected for survival on glucose. The fraction of glucose viable transformants was 26% in the G4-v4 based library and 4% in the wild-type library. Sequencing indicated that all 19 clones from the wild-type library carried lysine at 219. Variant G4-v4, however, accepted serine, lysine and five additional amino acids (Figure 4a).

The observed diversity in G4-v4 showed near-complete overlap with the natural diversity at position 219. The only exceptions were glycine that appeared viable in our selection (with the slowest growth rate; Figure 4b) but does not appear in known natural sequences, and leucine that appears in 5 out of the 73 archaeal PGKs included in the tree, but not in the G4-v4 sequenced clones. Indeed, when artificially introduced at position 219 of variant G4-v4, leucine did not confer any growth with glucose (data not shown).

### Dynamics of the mutational trajectories

**Figure 5:**
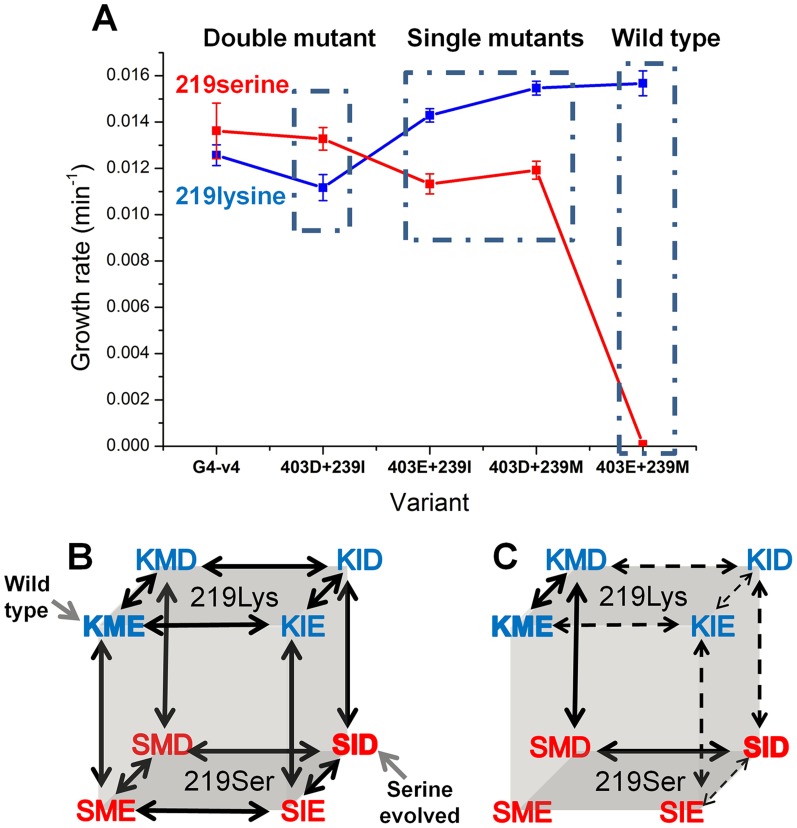
Epistatic effects of mutations underline divergence and incompatibility at position 219. (**a**) The engineered double mutant, I239M plus D304E, exhibits similar growth rate to the evolved variant G4-v4 with both lysine (in blue) and serine at 219 (in red). Thus, the 3 additional mutations in G4-v4 play a minor role (also indicated by I239M and D304E being the only mutations that were fixed; Table 1). Starting from the permissive state represented by the double mutant, both reversions, I239M and D304E, exhibit sign epistasis with respect to position 219: they improve growth at the background of lysine 219 but decrease growth with serine at 219. With respect to each other, M239I and E304D are nearly-additive at the background of lysine 219, but interact with strong negative epistasis at the background of serine 219. (**b**) All possible trajectories underlying the lysine-serine exchange. The first letter in each triplet represents position 219, the second represents 239, and the third, position 403. The wild-type and the K219S mutant are labeled. (**c**) Sign epistasis restricts the PGK accessible divergence routes. Under selection to maintain wild-type-like activity levels, no routes are accessible. Further, the few accessible routes under weak selection for PGK's activity (all arrows) boil down to a single route (solid arrows) under more stringent selection (growth rate ≤0.0125 min^−1^ vs. wild-type hPGK (0.0156 min^−1^)). Error bars represent S.D of 12 independent measurements. Note that a direct serine-lysine transition, i.e., by a single nucleotide exchange, is impossible. The most likely bridging intermediate is arginine, which seems to confer the highest growth rate at the background of the transition variant G4-v4 (Fig. 4b).

Can we deduce the order in which mutations that may enable exchanges at key positions such as 219 might occur? Two key mutations underlined the experimental, directed evolution towards the release of position 219 from serine incompatibility: M239I and E403D. To unravel a minimal trajectory that can lead to serine compatibility, the individual and cumulative effects of M239I and E403D were tested at the background of both lysine and serine at 219. The double mutant (M239I and E403D), either with lysine or serine at position 219, exhibited highly similar growth rates, and these were only slightly lower than those exhibited by the evolved transition variant G4-v4 (Figure 5a). The eight possible sequence permutations, including wild-type with either serine or lysine 219, were therefore constructed and tested for their growth rates. Analysis of the growth rates of the eight permutations indicates which trajectories are more likely to occur – these trajectories comprise ‘ridges’ in the genotype-phenotype space, i.e., the three mutational steps that connect the two fitness peaks, serine-219 and lysine-219 PGKs, with a minimal loss of fitness. These hypothetical trajectories indicate three trends (Figure 5b–c).

Firstly, the trajectory unraveled by our directed evolution experiment is underlined by strong sign epistasis. This trajectory can be referred to as a ‘reverse’ trajectory, namely from an extant lysine 219-restricted sequence (e.g. human PGK) to a 219-permissive sequence (the putative ancestral state). As predicted [6], both M239I and E403D, alone and in combination, exhibit a negative fitness effect in the context of lysine 219, but a positive effect when combined with serine 219. Not only does the sign revert, but the magnitude of fitness effects differs as well. However, the fitness gain at the background of serine 219 is ‘infinitely’ higher (from no growth to 0.013 min^−1^) relative to the loss at a lysine 219 background.

Secondly, the hypothetical ‘forward’ trajectory, i.e., a trajectory that might mimic the divergence of a 219-permissive sequence (the putative ancestral state) into the extant lysine-restricted sequences (e.g. human PGK), shows the same trend of sign epistasis. The mutations at 239 and 403 (to methionine 239 and glutamate 403, in the presumed forward direction) provide a small yet consistent fitness gain in the lysine 219 context (1.4-fold) yet result in a complete fitness loss on the background of serine 219. Indeed, this asymmetric effect is the underlining reason for the serine incompatibility of the extant lysine-219 PGK sequences.

Thirdly, although both mutations, I239M or D403E, contribute individually to serine-incompatibility (the hypothetical ‘forward’ direction), they interact with strong negative epistasis. The combination of both mutations completes the specialization towards lysine 219 and also makes hPGK incompatible to the serine exchange (no growth on glucose is observed with hPGK carrying serine 219).

The above eight permutations were also examined for growth in presence of the A397V mutation that appeared in round 6 and was subsequently removed in round 7 (see ‘sequence composition’ above). This analysis indicated that A397V is inferior to the E403D mutation in increasing the fitness of serine 219 (Figure S8). The combination of A397V and E403D showed very slow growth, that was much slower than that of E403D on its own. Thus, A397A and E403D, that never appeared together in a single variant, interact with negative epistasis. A397V therefore represents an alternative trajectory, albeit a trajectory that does not coincide with the optimal path and enforces a severe loss of fitness upon an exchange of lysine 219 to serine [7].

## Discussion

An ancient divergence event at the active site of an essential metabolic enzyme, PGK, provided us with a model for studying protein sequence divergence and incompatibility. The results of our laboratory evolution experiment indicate that as few as two mutations were sufficient to enable an exchange at a critical active site position that seems to have diverged with the archaeal lineage, presumably >3 billion years ago [33]. There are clear caveats associated with this experiment, and with the derived conclusions regarding the ‘fitness’ of individual PGK sequences and mutants. The laboratory conditions do not reproduce the range of environments and challenges that organisms face in the wild. Thus, the fact that under our experimental conditions, the growth rates with wild-type hPGK and its various mutants were similar to the parental strain with the endogenous, *E. coli* PGK, does not obviously imply the same fitness. Nonetheless, the experiment reveals a dramatic shift, from no growth on glucose in hPGK carrying serine 219, to near-normal laboratory growth upon acquiring the two rescuing mutations. The shift, however, occurred under two constraints.

Firstly, the trajectories enabling this exchange exhibit clear marks of sign epistasis, or indeed, of ‘reciprocal sign epistasis’ [7]- a large gain of function in the exchanged context (serine 219) is necessarily accompanied by a certain loss of fitness with respect to the original one (lysine 219). In other words, the fitness landscape is rugged and comprises two discrete peaks (serine or lysine 219), and all crossings between these peaks are via valleys of lower fitness (Figure 5b) [6]–[12]. However, our results also indicate that the valleys can be readily crossed. The exchanges at positions 219 and 403 are likely to have had a key contribution to the historical crossing, but substitutions at other positions that were not unraveled by our very limited exploration of PGK's sequence space may have further facilitated this crossing. An equally plausible explanation is that the historical crossing was enabled by a temporal relaxation in the selection pressure acting on PGK (Figure 5c). Relaxation may be afforded by an elevation in protein dose. Whereas the transition variant exhibited lower fitness than wild-type under limiting conditions (Figure 5a), this gap disappeared at ≥2-fold inducer level (AHT ≥15 ng/ml; data not shown). In nature, higher protein doses are readily obtained by promoter mutations [10] or by gene duplication [34]. Another enabling factor is a change in the environment, and specifically, alternative carbon sources. PGK is absolutely essential for growth in the presence of glucose, but growth rates of the evolved transition variants are far less compromised with additional carbon sources such as succinate and glycerol (Figure S5). Indeed, even highly essential metabolic processes can be bypassed by evolution [35].

Secondly, the exchange trajectory is highly specific in its sequence composition. Our selections were not bottlenecked and we sequenced a large variety of clones (110 in total). Nonetheless, two mutations, M239I and E403D, appeared in nearly all evolved variants. The mutation A397V comprises an alternative yet a transient solution, as it interacts with strong negative epistasis with the more optimal mutation E403D. There are also a number of other, global suppressor mutations that increase the activity upon exchange to serine, but their effects are relatively minor. Overall, the picture is consistent with the theory presented by Kondrashov *et al.* [6], and with previous findings regarding adaptive trajectories [10], [14]. That is to say, traversing sequence space at key, highly conserved positions can occur. However, the rate is painstakingly slow, primarily for two reasons: (*i*) The mutations that enable these key exchanges are highly specific, and thus the likelihood of traversing is low. (*ii*) Sign epistasis makes the accumulation of these enabling mutations dependent on a relaxation in the selection pressure, i.e., environmental changes or protein dose changes.

As we did not select for gain of function with any other amino acid than serine, the appearance of sequences that permit variability at position 219 was spontaneous, with respect not only to lysine, but to other amino acids as well. It seems that the two transition mutations, M239I and E403D, largely alleviated the enzyme's dependency on lysine, rather than having serine take over its role. Further, there is near-complete overlap between the amino acids tolerated by the evolved variant G4-v4 and those seen in archaeal PGKs (Figure 4). The sequence compositions isolated here, and particularly isoleucine at 239 and glutamate at 403, may therefore reflect the ancestral state with respect to position 219 at the node that separated the lysine-clade from the archaeal one. This node may represent a sub-optimal PGK yet with a unique capability to tolerate a wide range of amino acids at position 219.

Our results also show how specialization towards lysine (and probably towards any other amino acid seen in extant PGKs) erased the ancestral state with respect to an exchange in position 219. Thus, while hPGK may have been reverted to an ancestral state with respect to position 219, this reversion depended on a rare combination of at least two mutations in other sites [36]. The challenge of reverting relates to the fact that the mutations that augment G4-v4's activity with lysine severely compromised activity with serine. This challenge is also manifested in the need to implement a special protocol that blocks the reversion to lysine in the laboratory evolution experiment. Needless to say that such blocks are impossible in natural evolution. Foremost, a strong negative epistatic effect seems to have led to the incompatibility seen in the extant human PGK (Figure 5). Thus, incompatibility may not be the outcome of drift, as in the classical Dobzansky-Muller model for hybrid incompatibility [37], [38]. Rather, incompatibility is the outcome of the evolution towards higher performance with lysine-219, and the highly deleterious effect of the mutations that improve the activity with lysine-219 at the background of serine-219.

From a sequence space point of view, this work reinforces the view that “functional proteins must form a continuous network which can be traversed by unit mutational steps without passing through nonfunctional intermediates” [19]. The experimental approach taken here provides crucial insights in this respect. For example, a lysine-serine exchange cannot occur via a single nucleotide substitution. Thus, an intermediary amino acid must have existed at some stage. Ancestral inference does not reveal possible candidates, as the predicted nodes carry at position 219 either lysine (the LUCA's PGK) or serine (the ancestor of archaeal PGKs). However, the screen for tolerable amino acids at position 219 of the transition variant G4-v4's revealed that the growth rate with arginine exceeded all other amino acids including lysine (Figure 4b). Indeed, arginine does comprise a bridging codon between serine and lysine (Figure S9). Thus, the experiment reveals that arginine is the most probable transition between lysine and serine in the corresponding PGK clades.

From a protein structure-function point of view, our results support a somewhat unorthodox view: some sites are conserved not because a given amino acid is functionally irreplaceable (e.g. lysine 219 of human PGK), but rather because “there has not been enough time to create the right combination of amino acids at other sites to allow them to evolve” [6]. The structural and functional features that underline the exchange at position 219 are unknown at this stage, but the evolutionary basis of this particular exchange seems to have been clarified.

## Materials and Methods

Detailed protocols are provided as supplemental methods.

### Phylogenetic analysis

PGK sequences were collected by combining three BLAST searches using human, *E. coli* and *M. mazei* PGK sequences as queries. Each of the three BLAST searches contained sequences from all three kingdoms of life, suggesting that the searches were exhaustive. Glycerate kinase from *Neisseria meningitides* was structurally aligned with the PGK sequences to serve as an outgroup, in agreement with the protein fold classification [39]. The sequences were aligned using MUSCLE [40], and ≥98% redundancy was removed using CDHIT [41]. A phylogenetic tree was built using PHYML [42] and the JTT substitution matrix. The tree was visualized with FIGTREE. As observed in a tree of 131 PGK sequences [20], the three kingdoms formed distinct clades. However, the inter-kingdom topology deviates from the species tree [43] whereby the eukaryotic branch diverges from within the bacterial branch with the spirochaetes being the outgroup. The tree and the alignment were further processed by PAML [44] for ancestral sequence prediction using default parameters.

### Directed evolution

The *pgk* knockout strain was constructed using the technique of Datsenko and Wanner [45] with primers PGK_KO_F and PGK_KO_R. A set of plasmids with decreasing copy numbers were constructed by modifying pZA11MCS (EXPRESSYS) as described in Text S1 (Figure S12). To generate the library for the first round of directed evolution, the K219S mutant of hPGK was amplified by error-prone PCR (GeneMorph II Random Mutagenesis Kit; Agilent) at an average of 2.5±2 mutations per gene. The resulting gene library was re-cloned to pZUC (the K219S mutant did not grow on media containing glucose when expressed from the low copy pZA plasmid; Figure 2). The growth upon copy number manipulation correlated with very high protein expression levels (Figure S10). The resulting plasmid library was transformed into Δ*pgk* cells and plated on minimal media agar plates supplemented with 20 mM glucose, kanamycin and ampicillin (selection plates). An aliquot of the transformation reaction was plated on glycerol-succinate plates with both antibiotics to measure the transformation efficiency. The colonies that grew on the selection plates were pooled and the plasmid DNA was isolated. Prior to pooling, 10 to 20 variants were sequenced from randomly picked clones. The hPGK gene was amplified from the plasmid pool by using the special cloning protocol designed to eliminate mutations at position 219 (Figure S11). The PCR product was digested by Nco1 and Not1, purified from gel and subsequently cloned to the appropriate plasmid for the next round of selection. In the 2^nd^ round, gene shuffling was applied to obtain favorable combinations of mutations that accumulated in the 1^st^ round (see Text S1). In later rounds, mutagenesis was achieved via the use of 60 cycles of non-proofreading TAQ polymerase. The subsequent rounds (R2 to R7) were conducted with certain modifications to allow an increase in selection pressure: (a) Use of lower copy plasmids; (b) lower inducer (AHT) concentration in the selection plates; (c) shorter time of incubation of the selection plates. A summary of the selection conditions for all rounds is provided as Table 2.

### Characterization of individual variants

Representative variants from the selected libraries were chosen for further analysis. The variants were chosen so they reflect the mutational trend within their round, and order in which mutations fixed in course of the progressing generations from G1 to G7. For instance, the E403D mutation already appeared in 3 out of 9 clones that were sequenced from the first round library, and this mutation was enriched in consecutive rounds. Thus, the chosen variant G1-v5 carried this mutation (Table 1). Conversely, the M239I mutation became dominant (≥30% of the clones) only by the third round, and the combination of both mutations in a single clone represents the general trend in the fourth round library, as represented in variant G4-v4. In round 6, the A397V mutation became enriched (found in 35% of the sequences variants) and appeared to be mutually exclusive from the E403D mutation. Thus, variant G6-v9 was chosen as a representative sequence. We also assumed that mutations that appeared only once in the sequenced variants from all rounds, did not contribute to the enhancement of hPGK- serine 219's activity. Thus, another criterion for choosing variants for analysis was a minimal number of ‘hitchhiking’ mutations. Finally, the growth rates, kinetic parameters and sequence compositions of 12 additional variants are given as Table S2 – these coincide with the variants presented in the main text. The chosen variants were re-cloned to pZA. For each clone, a lysine 219 counterpart was prepared by all-around PCR with primers S219K_F and S219K_R. Mutations M239I and E403D were similarly incorporated (primers listed in Table S3). Plasmids encoding individual hPGK variants were transformed into Δ*pgk* cells and the sequences were revalidated. Single colonies were used to inoculate 2 ml of glycerol-succinate media with kanamycin and ampicillin. The OD^600 nm^ were measured, and fresh glycerol-succinate media supplemented with 5 mM glucose and 8 ng/ml AHT were inoculated with these overnight cultures at the same initial bacterial density (OD^600 nm^ = 0.02). The inoculated media (200 µl) were distributed in random fashion in a 96 well-plate. The plate was incubated at 37°C, and 95% relative humidity, in a rotating incubator (Liconic Instruments, Woburn, MA). Using an automated robotic platform (Evoware II, Tecan) and a multi-well reader (Infinite M200-pro, Tecan) the OD^600 nm^ was measured at 20 min intervals. The growth rate was calculated by plotting the log2 of the OD^600 nm^ against time of incubation, and calculating linear regression values over a window size of 2 hours. The 3 highest rates (ΔOD/Δt values) along the entire growth curve were extracted and averaged. Doubling time was calculated as the reciprocal of the average growth rate [46]. Error bars correspond to standard errors from 8 independent measurements of each variant.

## Supporting Information

Figure S1 A schematic representation of PGK's active site with its substrates: ADP, 3-phosphoglycerate and the transition state analog (MgF_3_^−^). In bold are residues which are completely conserved throughout the alignment. Highlighted in red is the catalytic residue lysine 219. All eukaryotic and prokaryotic PGKs possess a 336-asparagine. Most archaeal sequences possess an asparagine, with serine, lysine and arginine sporadically appearing in this clade. The N336S mutation in PGK had a similar diminishing effect as K219S, both on growth and in kinetic parameters (Figure S3). The scheme was prepared using the published transition state analog complex of human PGK (PDBid 2WZB). ^a^ Residues that are conserved only among eukaryotic and prokaryotic PGKs. ^b^ residues that have drifted significantly and do not show any pattern of conservation
(TIF)

Figure S2 Three dimensional scheme of PGK's active site. Shown are lysine 219, ADP, 3-phosphoglycerate (3PG) and the transition state analog (AlF_4_^−^) in stick representation. Indicated are the distances between lysine 219's amine to ADP's α-phosphate's oxygen and the fluoride of AlF_4_^−^ which represents the transferred phosphate group. The Figure was prepared using PDB structure 2YBE.
(TIF)

Figure S3 Michaelis-Menten curves. (a) Catalytic efficiency of wild type PGK ([E]_0_ = 8 nM, squares), PGK- serine 219 ([E]_0_ = 0.12 µM, triangles) and PGK- serine 336 ([E]_0_ = 0.1 µM, circles). (b) Focused measurements of the linear areas of the plots. The fit of the linear regions of the plots is of initial rates (υ_0_) versus initial substrate concentrations ([S]_0_). The slopes were used to calculate *k_cat_/K_M_* values since PGK is activated by the substrate anion and therefore displays complex kinetics [47].The *k_cat_/K_M_* for wild type PGK was in disagreement with previously reported measurements [18].
(TIF)

Figure S4 Lysine to glutamate surface mutations that were abundant in round 7. All of the shown seven mutations appeared in more than a single variant within the 7^th^ round. The figure was prepared using PDB structure 2X13.
(TIF)

Figure S5 Growth rates of PGK variants with alternative carbon sources. Growth rates of wild type PGK, the K219S mutant, the evolved variants and their lysine counterparts, on either glycerol-succinate media supplemented with 5 mM glucose (solid lines), or on 5 mM glucose as sole carbon source (dashed lines). All other conditions remained identical.
(TIF)

Figure S6 Trees depicting the phylogenetic distribution of positions 239 and 403. Both trees were prepared similarly to the tree shown in figure 1. All PGKs in the Bacteria/Eukaryotes clade posses a 219 lysine while the Archaeal PGKs never have a 219 lysine. (a) PGK sequences with a 403 glutamate are colored in cyan and those that do not have a 403 glutamate (mostly alanine) are in black. (b) PGK sequences with a 239 methionine are colored in orange and those that do not have a 239 methionine (mostly valine) are in black.
(TIF)

Figure S7 Phylogenetic distribution of residues occupying position 219 within archaeal PGKs. A schematic phylogenetic tree of 75 archaeal PGKs rooted by a eukaryotic PGK from *S. cerevisiae*. Colors represent amino acid occupying position 219 (black – serine, blue – threonine, green – valine, yellow – leucine, red – alanine and cyan – arginine).
(GIF)

Figure S8 All permutations with the A397V mutation. Growth rates of all 16 mutational permutations including the A397V mutation. For each permutation the lysine 219 variant is shown in blue and the serine 219 in red.
(TIF)

Figure S9 All possible transitions between lysine and serine via single nucleotide exchange steps. Arg, Thr and Asn are the only candidates. Arg and Thr were selected from the 219-diversified library based on G4-v4, with Arg being the fittest variant making it the most probable ancestral state at the split between the archaeal and the bacteria/eukaryotic branches.
(TIF)

Figure S10 Plasmid copy number dramatically affects PGK expression levels. Shown is an SDS-PAGE analysis of Δ*pgk* cells complemented with either pZA-hPGK Serine 219 (left side) grown with 20 ng/ml AHT or pZUC-hPGK Serine 219 grown with no inducer. S and P stand for soluble fraction and pellet, respectively. The large band seen in the lane representing the soluble fraction of pZUC (depicted with a black arrow), corresponds to the size of hPGK.
(TIF)

Figure S11 Schematic description of the cloning protocol for preventing mutations at position 219. (a) Two separate PCR reactions are conducted using the plasmid pool from the selected clones as template, with primers pZ_F and MF_R (left fragment), and with primers pZ_R and MF_F (right fragment, primers are represented by black arrows; primers pZ_F and pZ_R are depicted by an asterisk). The primers revert any changes in position 219 to serine, and the original plasmid template is destroyed by digestion with DpnI. The red rectangles represent the Bpm1 recognition sites. The purified PCR products were digested with Bpm1 (NEB) and purified from agarose gel. Upon digestion with Bpm1, the flanking segments between the Bpm1 recognition sites and the digestion sites are eliminated. These sequences originate from the MF primers, and thus might result in reversion of mutations that have accumulated in previous rounds. (b) The two gene segments are liagted, and the intact gene is obtained by amplification with primers pZ_F and pZ_R. Digestion with Nco1 and Not enables re-cloning into the selection plasmid. (c) Zoom-in view on the nucleotide sequence of the MF primers within the double stranded PCR product. The black arrows depict the directionality from 5′ to 3′. The serine codon is shown in bold and the Bpm1 recognition (5′-CTGGAG-3′) site is in a red box. (d) Upon Bpm1 restriction, the DNA is digested away from the recognition sequence to create the sticky ends for ligation, thus restoring the serine codon. The digestion products that are further purified, ligated and amplified are shown in blue boxes.
(TIF)

Figure S12 A map of the pZA plasmid containing *pgk* used for the directed evolution and for measuring the growth rates of PGKs. The ampicillin resistance gene and the TET repressor gene are constitutively expressed from the same promoter forming a single mRNA. The pZUC and pZE plasmids were prepared by replacing the p15A origin of replication with the pUC and colE1 origins, respectively.
(TIF)

Table S1 Plasmids used for the directed evolution experiment.
(DOCX)

Table S2 All variants underlining the directed evolution of hPGK- serine 219. Noted are sequence changes, growth rates and *k_cat_/K_M_* values of representative variants along the laboratory trajectory. Variants annotation: G stands for the round number from which a variant was isolated, and v denotes the variant's sequential number within this round. The left column denotes the sequence composition and rate parameters of wild-type hPGK. Variants appearing in bold are the representative variants shown in Table 1 and in Figure 3.
(XLSX)

Table S3 List of oligonucleotides and primers used in this study (from 5′ to 3′).
(DOCX)

Text S1 Supplementary information.
(DOCX)
