# Supplementary figures and images for: Mechanisms of Protein Sequence Divergence and Incompatibility
Source: PLoS Genet. 2013 Jul 25;9(7):e1003665. doi: 10.1371/journal.pgen.1003665 (PMC3723536; doi:10.1371/journal.pgen.1003665)

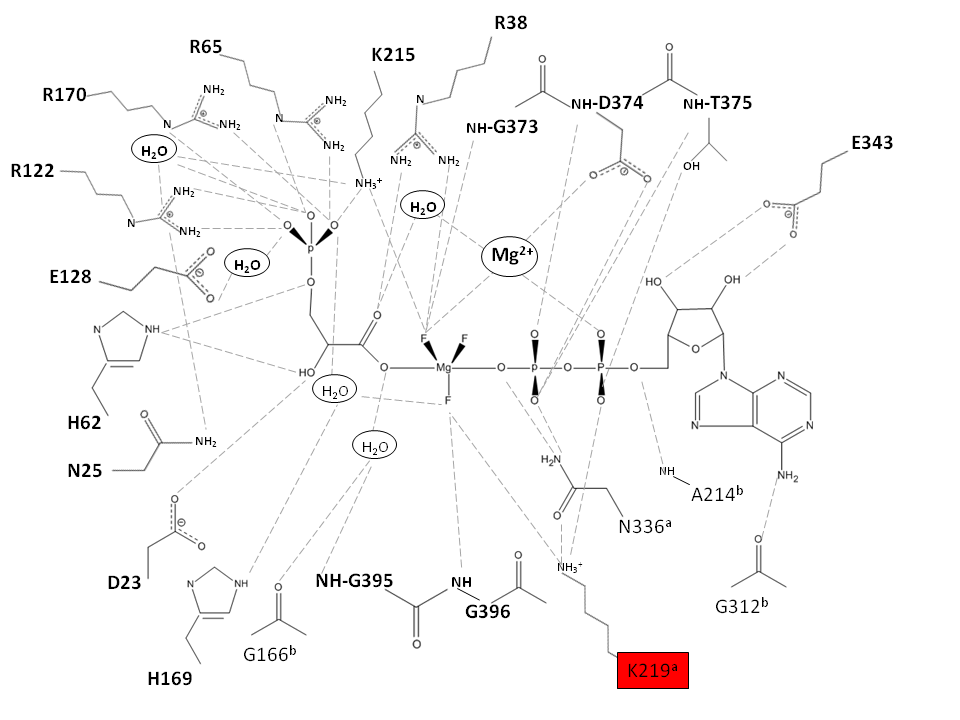

Supplement: Figure S1 — A schematic representation of PGK's active site with its substrates: ADP, 3-phosphoglycerate and the transition state analog (MgF3 −). In bold are residues which are completely conserved throughout the alignment. Highlighted in red is the catalytic residue lysine 219. All eukaryotic and prokaryotic PGKs possess a 336-asparagine. Most archaeal sequences possess an asparagine, with serine, lysine and arginine sporadically appearing in this clade. The N336S mutation in PGK had a similar diminishing effect as K219S, both on growth and in kinetic parameters (Figure S3). The scheme was prepared using the published transition state analog complex of human PGK (PDBid 2WZB). a Residues that are conserved only among eukaryotic and prokaryotic PGKs. b residues that have drifted significantly and do not show any pattern of conservation (TIF) [file pgen.1003665.s001.tif]

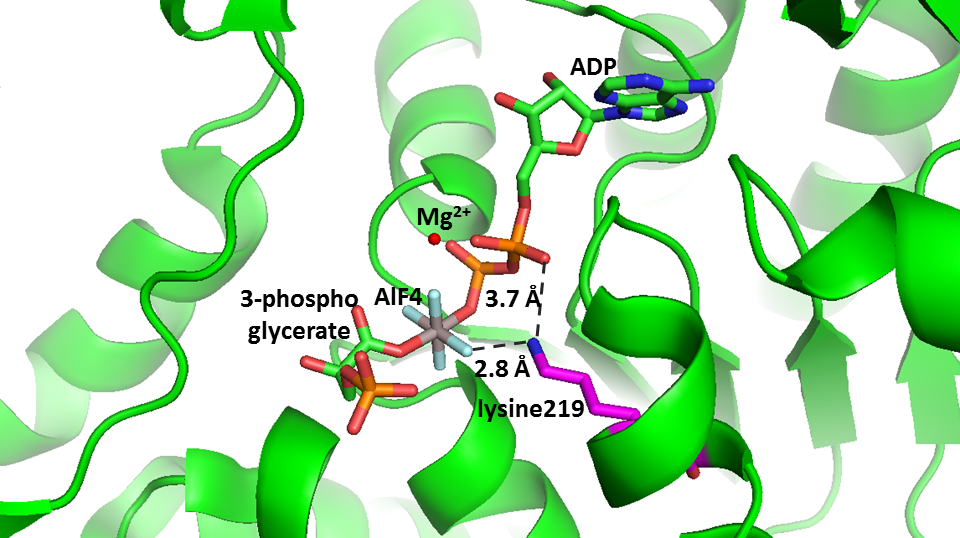

Supplement: Figure S2 — Three dimensional scheme of PGK's active site. Shown are lysine 219, ADP, 3-phosphoglycerate (3PG) and the transition state analog (AlF4 −) in stick representation. Indicated are the distances between lysine 219's amine to ADP's α-phosphate's oxygen and the fluoride of AlF4 − which represents the transferred phosphate group. The Figure was prepared using PDB structure 2YBE. (TIF) [file pgen.1003665.s002.tif]

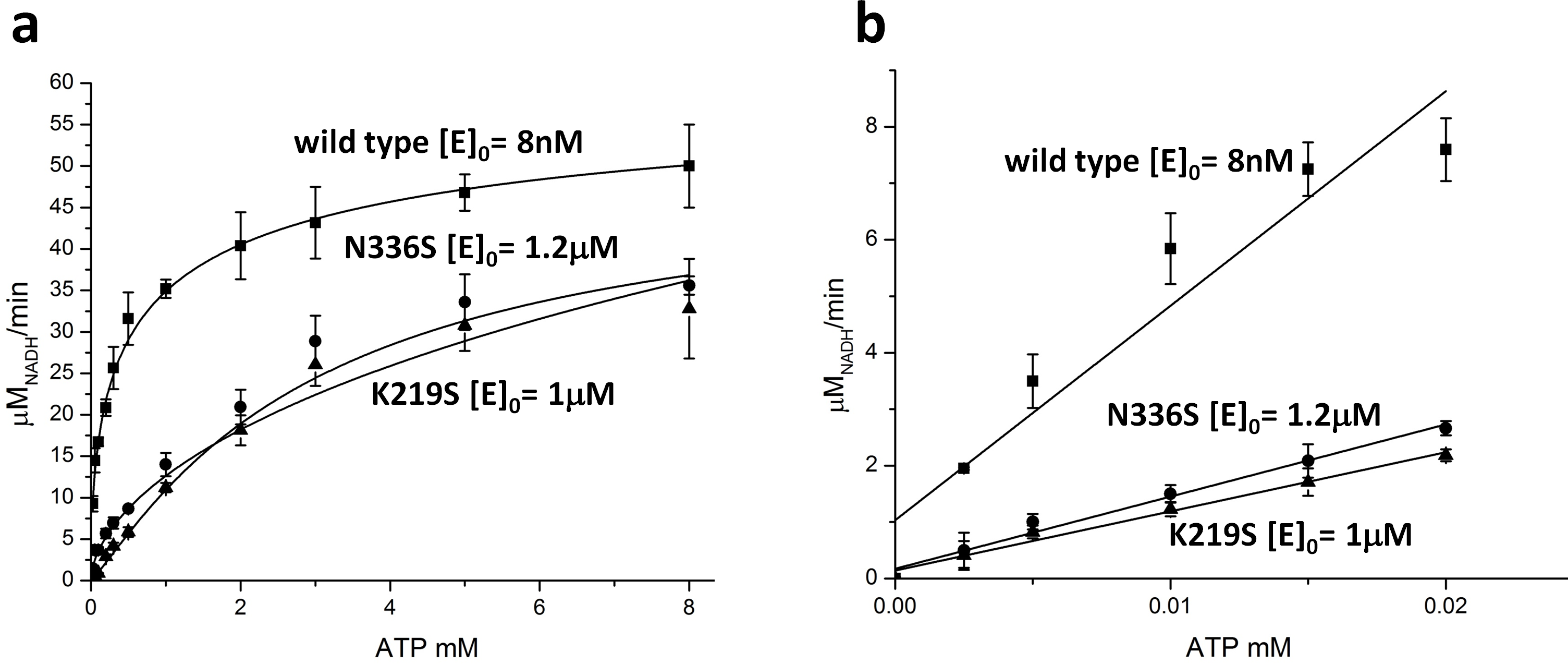

Supplement: Figure S3 — Michaelis-Menten curves. (a) Catalytic efficiency of wild type PGK ([E]0 = 8 nM, squares), PGK- serine 219 ([E]0 = 0.12 µM, triangles) and PGK- serine 336 ([E]0 = 0.1 µM, circles). (b) Focused measurements of the linear areas of the plots. The fit of the linear regions of the plots is of initial rates (υ0) versus initial substrate concentrations ([S]0). The slopes were used to calculate kcat/KM values since PGK is activated by the substrate anion and therefore displays complex kinetics [47].The kcat/KM for wild type PGK was in disagreement with previously reported measurements [18]. (TIF) [file pgen.1003665.s003.tif]

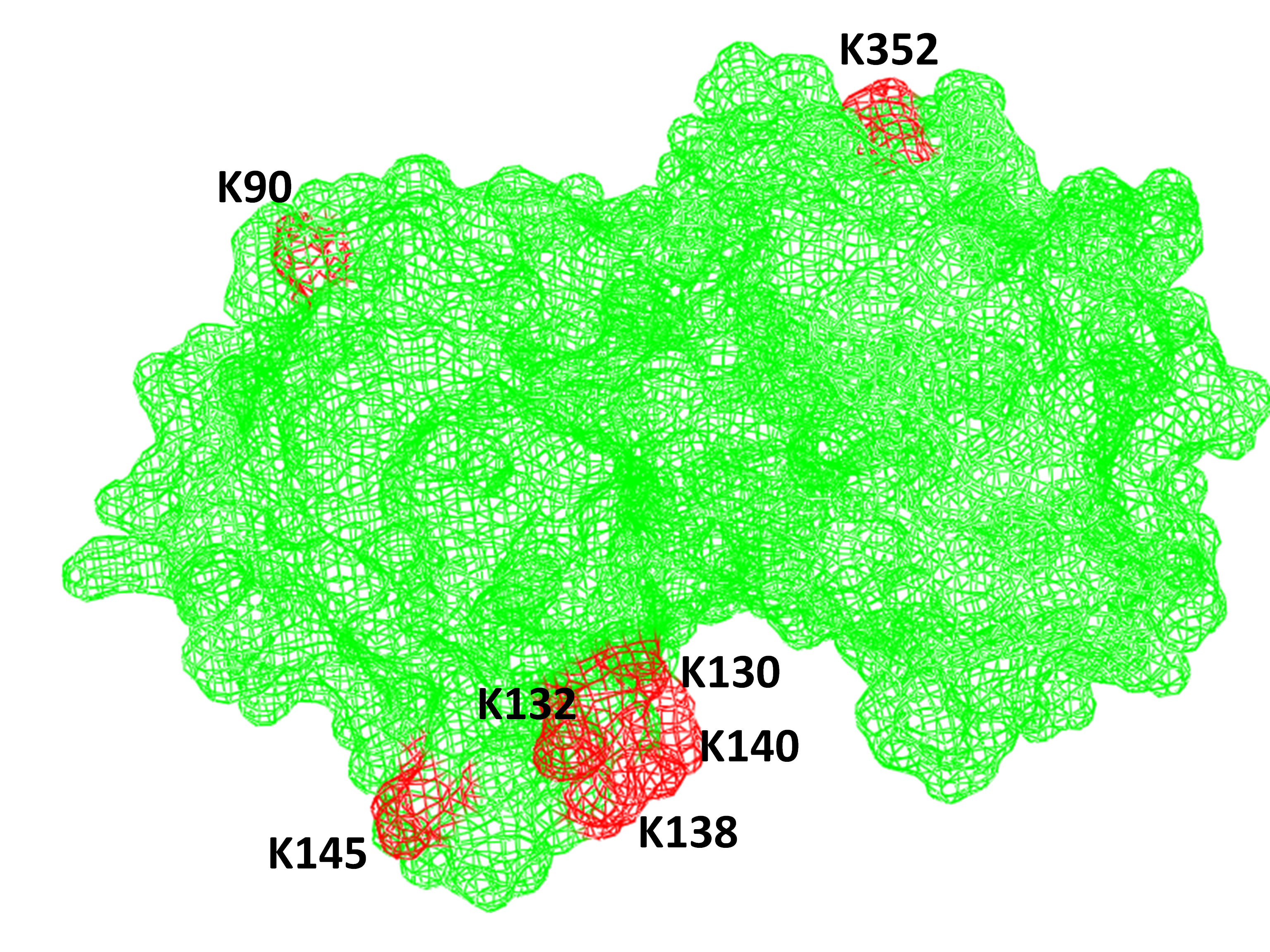

Supplement: Figure S4 — Lysine to glutamate surface mutations that were abundant in round 7. All of the shown seven mutations appeared in more than a single variant within the 7th round. The figure was prepared using PDB structure 2X13. (TIF) [file pgen.1003665.s004.tif]

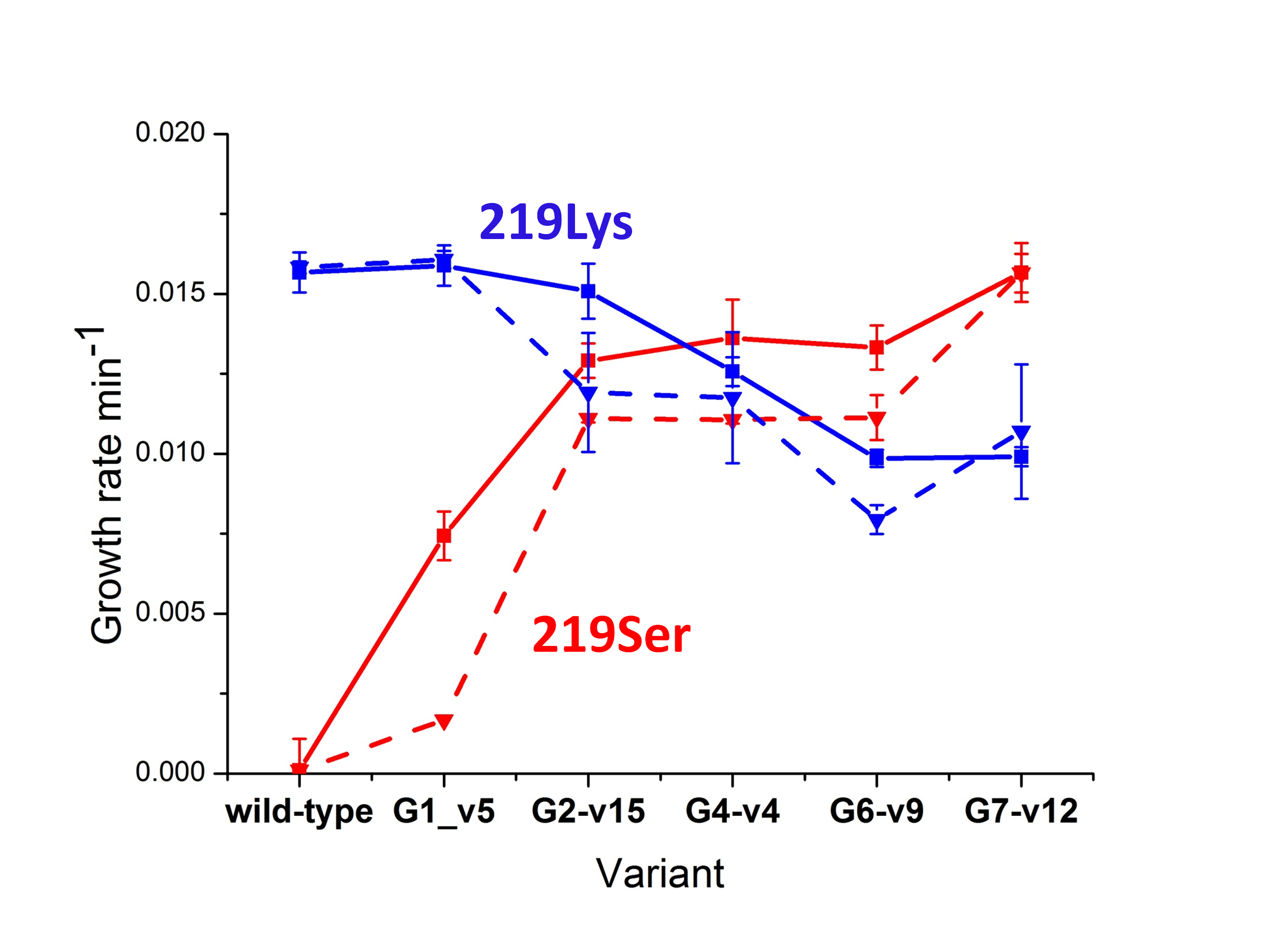

Supplement: Figure S5 — Growth rates of PGK variants with alternative carbon sources. Growth rates of wild type PGK, the K219S mutant, the evolved variants and their lysine counterparts, on either glycerol-succinate media supplemented with 5 mM glucose (solid lines), or on 5 mM glucose as sole carbon source (dashed lines). All other conditions remained identical. (TIF) [file pgen.1003665.s005.tif]

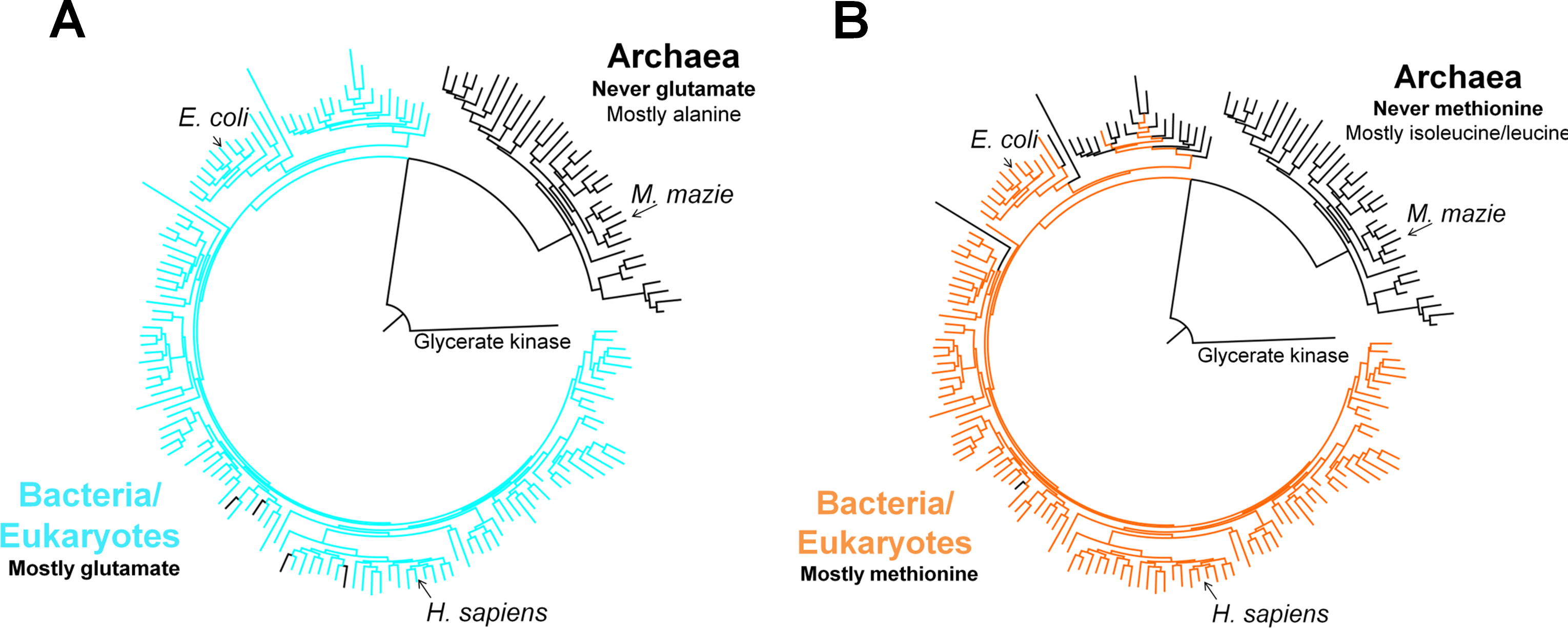

Supplement: Figure S6 — Trees depicting the phylogenetic distribution of positions 239 and 403. Both trees were prepared similarly to the tree shown in figure 1. All PGKs in the Bacteria/Eukaryotes clade posses a 219 lysine while the Archaeal PGKs never have a 219 lysine. (a) PGK sequences with a 403 glutamate are colored in cyan and those that do not have a 403 glutamate (mostly alanine) are in black. (b) PGK sequences with a 239 methionine are colored in orange and those that do not have a 239 methionine (mostly valine) are in black. (TIF) [file pgen.1003665.s006.tif]

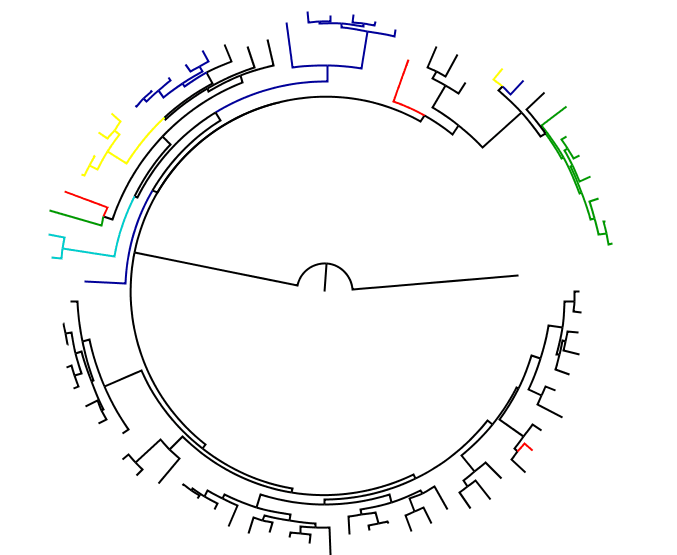

Supplement: Figure S7 — Phylogenetic distribution of residues occupying position 219 within archaeal PGKs. A schematic phylogenetic tree of 75 archaeal PGKs rooted by a eukaryotic PGK from S. cerevisiae. Colors represent amino acid occupying position 219 (black – serine, blue – threonine, green – valine, yellow – leucine, red – alanine and cyan – arginine). (GIF) [file pgen.1003665.s007.gif]

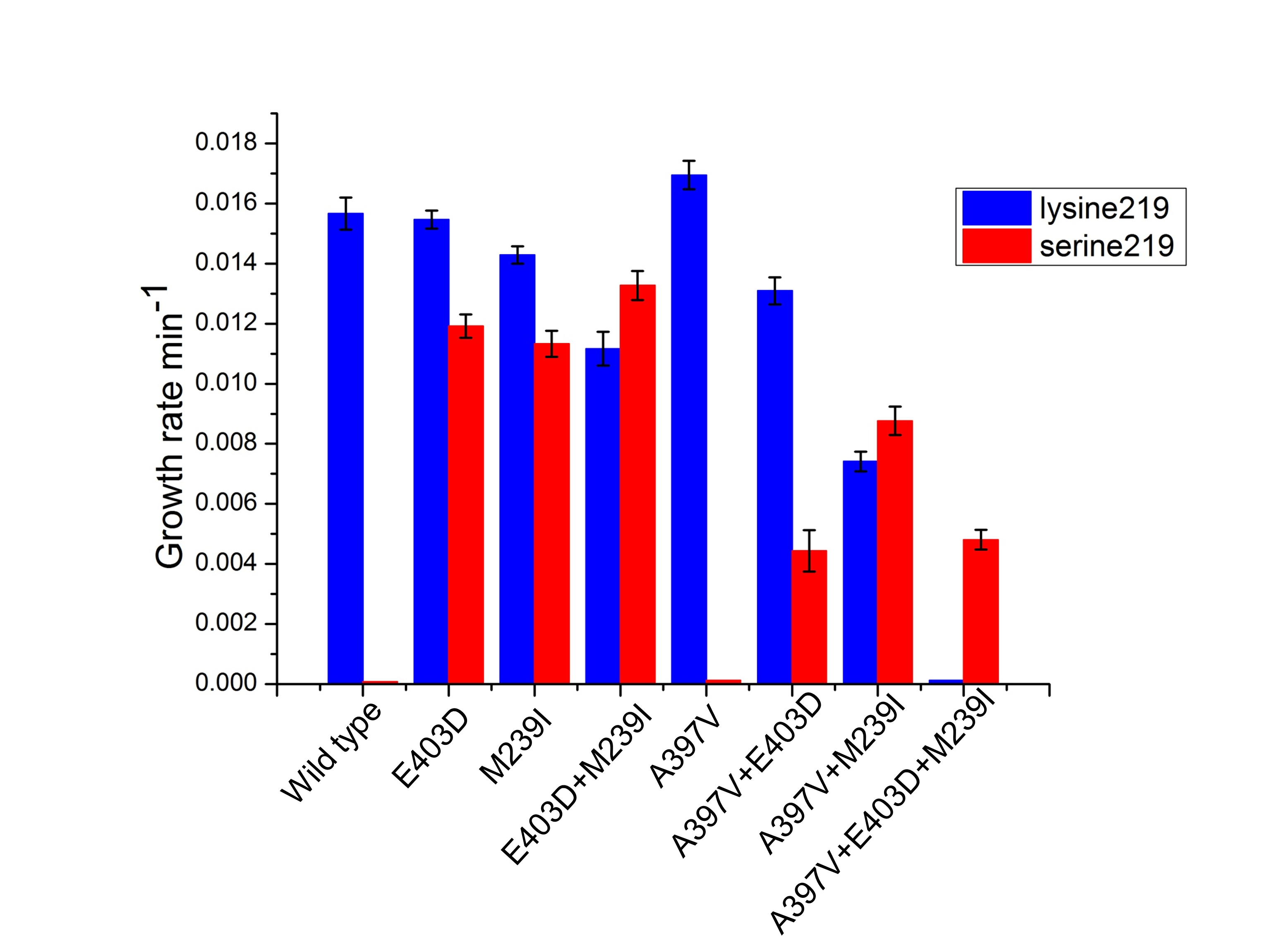

Supplement: Figure S8 — All permutations with the A397V mutation. Growth rates of all 16 mutational permutations including the A397V mutation. For each permutation the lysine 219 variant is shown in blue and the serine 219 in red. (TIF) [file pgen.1003665.s008.tif]

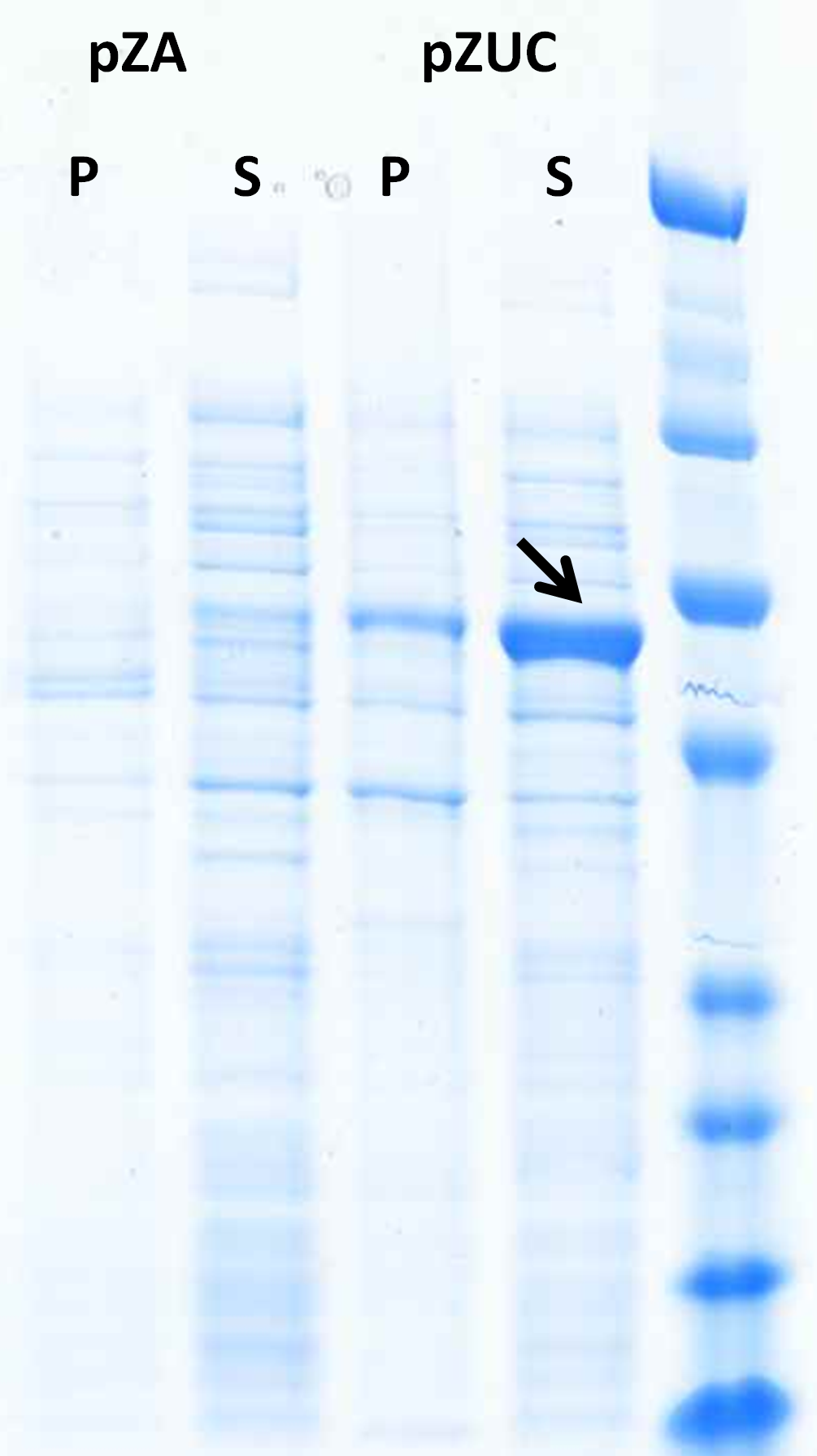

Supplement: Figure S10 — Plasmid copy number dramatically affects PGK expression levels. Shown is an SDS-PAGE analysis of Δpgk cells complemented with either pZA-hPGK Serine 219 (left side) grown with 20 ng/ml AHT or pZUC-hPGK Serine 219 grown with no inducer. S and P stand for soluble fraction and pellet, respectively. The large band seen in the lane representing the soluble fraction of pZUC (depicted with a black arrow), corresponds to the size of hPGK. (TIF) [file pgen.1003665.s010.tif]

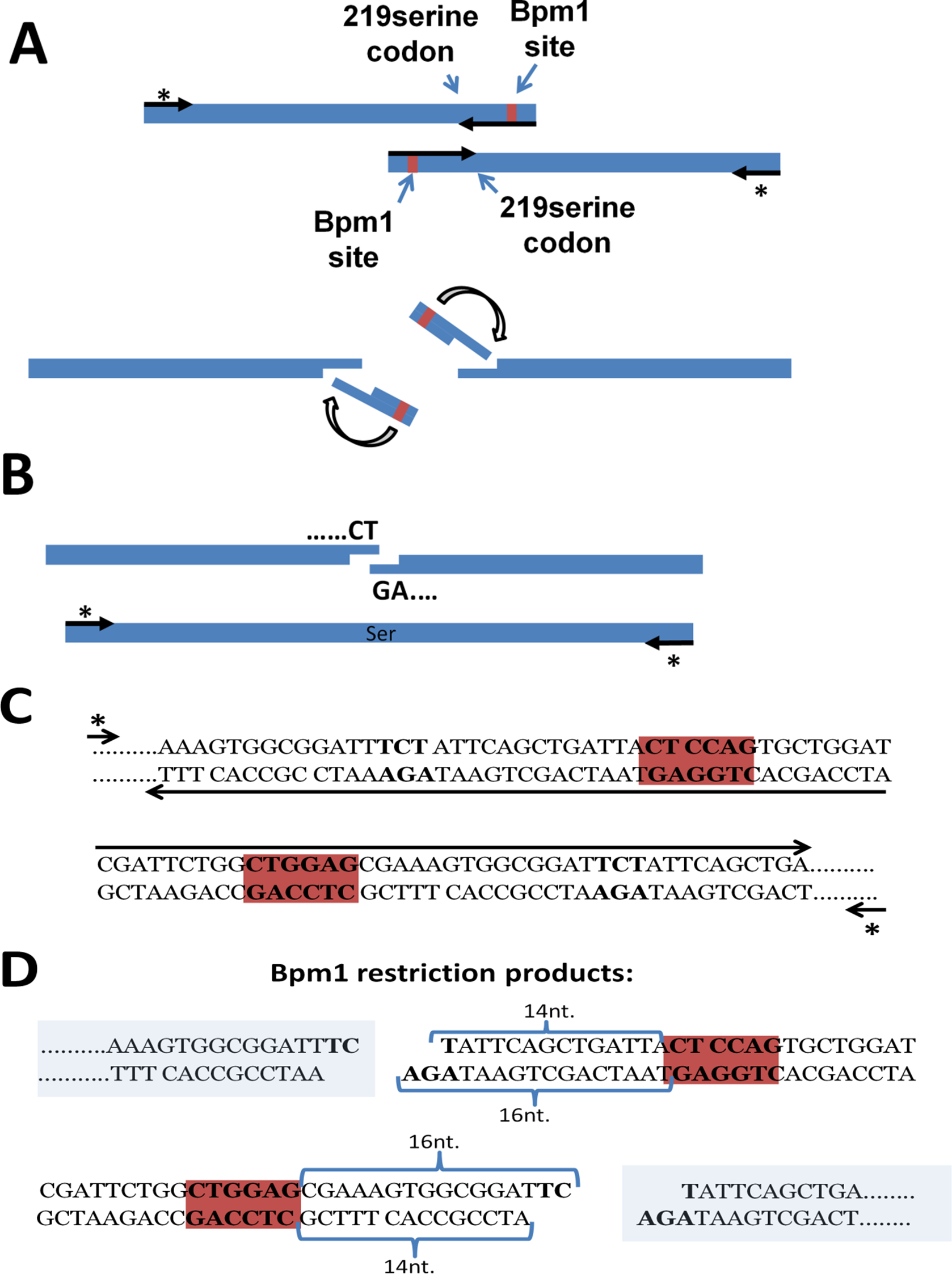

Supplement: Figure S11 — Schematic description of the cloning protocol for preventing mutations at position 219. (a) Two separate PCR reactions are conducted using the plasmid pool from the selected clones as template, with primers pZ_F and MF_R (left fragment), and with primers pZ_R and MF_F (right fragment, primers are represented by black arrows; primers pZ_F and pZ_R are depicted by an asterisk). The primers revert any changes in position 219 to serine, and the original plasmid template is destroyed by digestion with DpnI. The red rectangles represent the Bpm1 recognition sites. The purified PCR products were digested with Bpm1 (NEB) and purified from agarose gel. Upon digestion with Bpm1, the flanking segments between the Bpm1 recognition sites and the digestion sites are eliminated. These sequences originate from the MF primers, and thus might result in reversion of mutations that have accumulated in previous rounds. (b) The two gene segments are liagted, and the intact gene is obtained by amplification with primers pZ_F and pZ_R. Digestion with Nco1 and Not enables re-cloning into the selection plasmid. (c) Zoom-in view on the nucleotide sequence of the MF primers within the double stranded PCR product. The black arrows depict the directionality from 5′ to 3′. The serine codon is shown in bold and the Bpm1 recognition (5′-CTGGAG-3′) site is in a red box. (d) Upon Bpm1 restriction, the DNA is digested away from the recognition sequence to create the sticky ends for ligation, thus restoring the serine codon. The digestion products that are further purified, ligated and amplified are shown in blue boxes. (TIF) [file pgen.1003665.s011.tif]

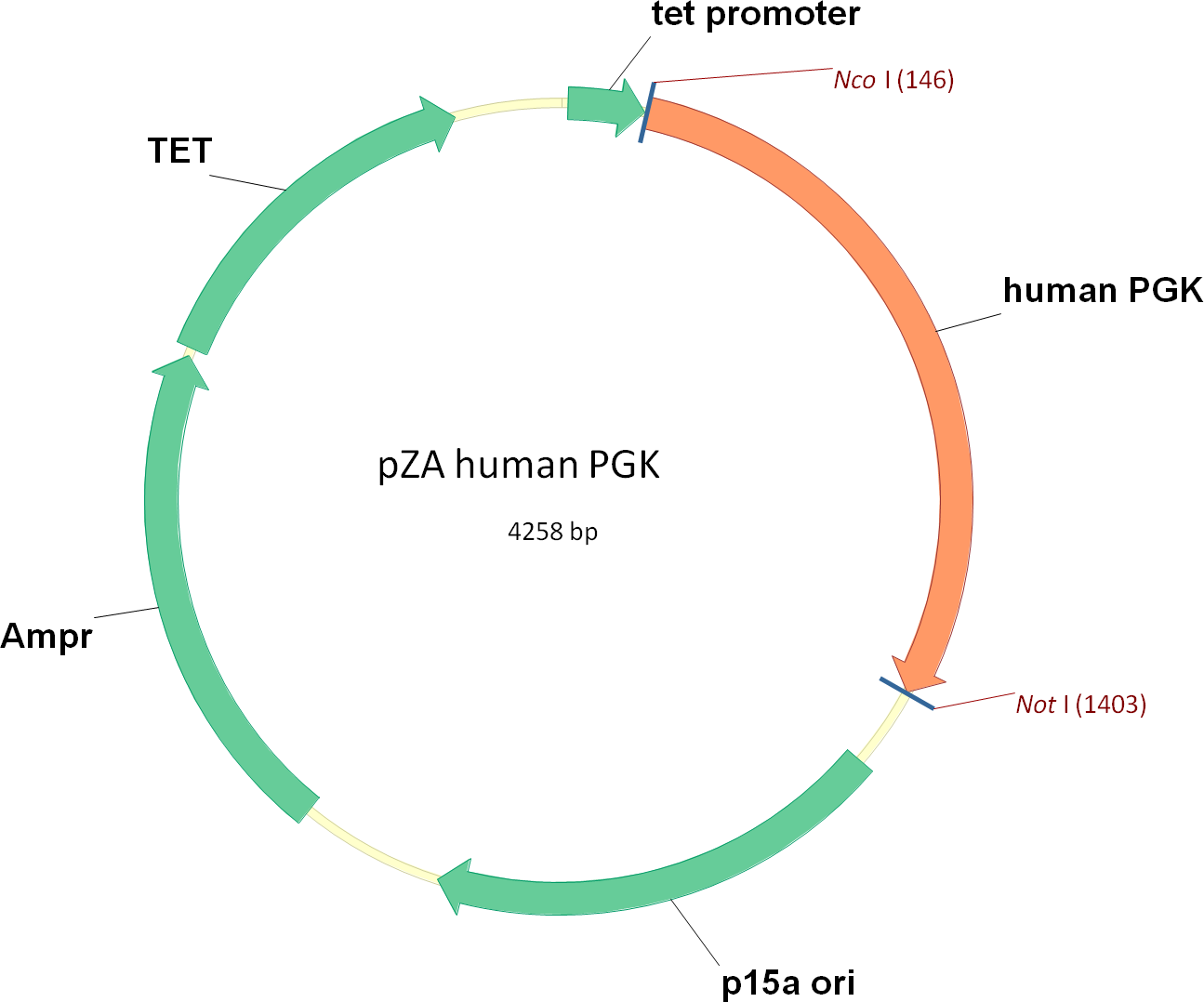

Supplement: Figure S12 — A map of the pZA plasmid containing pgk used for the directed evolution and for measuring the growth rates of PGKs. The ampicillin resistance gene and the TET repressor gene are constitutively expressed from the same promoter forming a single mRNA. The pZUC and pZE plasmids were prepared by replacing the p15A origin of replication with the pUC and colE1 origins, respectively. (TIF) [file pgen.1003665.s012.tif]
