# Supplementary material for: Mechanisms of Protein Sequence Divergence and Incompatibility
Source: PLoS Genet. 2013 Jul 25;9(7):e1003665. doi: 10.1371/journal.pgen.1003665 (PMC3723536; doi:10.1371/journal.pgen.1003665)
Supplement: Table S1 — Plasmids used for the directed evolution experiment. (DOCX) [file pgen.1003665.s013.docx]

| plasmid | pZUC | pZE | pZA |
| --- | --- | --- | --- |
| Origin of replication | pUC | colE1 | p15A |
| Copies per cell | ~900 [6] | 70-100 [7] | 30-50 [7] |
