# Supplementary material for: Mechanisms of Protein Sequence Divergence and Incompatibility
Source: PLoS Genet. 2013 Jul 25;9(7):e1003665. doi: 10.1371/journal.pgen.1003665 (PMC3723536; doi:10.1371/journal.pgen.1003665)
Supplement: Table S3 — List of oligonucleotides and primers used in this study (from 5′ to 3′). (DOCX) [file pgen.1003665.s015.docx]

| PGK_KO_F | CGCAAGCAGCGTCTGCAAAACTTTTAGAATCAACGAGAGGATTCACCATGA  TTCCGGGGATCCGTCGACC |
| --- | --- |
| PGK_KO_R | AAG GGCAGGTTTCCCTGCCCTGTGATTTTTTACTTCTTAGCGCGCTCTTCTGT  AGGCT GGAG CTGCTTCG |
| DEL_VER_F | CGATCCGCACAGTGC CATTGTCG |
| DEL_VER_R | ATCGTCGGGCCTTATACTCGTCAT |
| K219S_F | TCTATTCAGCTGATT AACAACATGC |
| K219S_R | ATCCGCCACTTTCGCGCCGCCC |
| N336S_F | GATTGTGTGGTCGGGCCCGGT |
| N336S_R | CCGGGCCCGACCACACAATC |
| MTM_S219K_F | CGAAAGTGGATGATAAACTGCGCGTGACCG |
| MTM_S219K_R | CGGTCACGCGCAGTTTATCATCCACTTTCG |
| pZ_F | CTCGAGTCCCTATCAGTGAT |
| pZ_R | CCCTAGGTCTAGGGCGGCGG |
| NSTD_F | CCTAATTTTTGTTGACACTC |
| NSTD_R | GCGCCATCTCCTGCAAGCTT |
| MF_F | TTTATCCAGCACTGGAGTAATCAGCTGAATAGA |
| MF_R | CGATTCTGGCTGGAGCGAAAGTGGCGGATTCT |
| S219K_F | AAAATTCAGCTGATTAACAACATGCTGGATAAA |
| S219K_R | ATCCGCCACTTTCGCGCCGCCCAGAATCGCC |
| NNS_F | NNSATTCAGCTGATTAACAACATGCTGGATAAA |
| M239I_F | CGAAATGATTATTGGCGGCGGCATAGCGTTTACCTTTCTGAAAGTGCTGAA  CAACATGG |
| M239I_R | CCATGTTGTTCAGCACTTTCAGAAAGGTAAACGCTATGCCGCCGCCAATAA  TCATTTCG |
| E403D_F | CCGGCGGCGGCGCGAGCCTGGAACTGCTGGATGGCAAAGTGCTGCCGGG  CGTGGATGCGC |
| E403D_R | GCGCATCCACGCCCGGCAGCACTTTGCCATCCAGCAGTTCCAGGCTCGCGC  CGCCGCCGG |
| A397V_F | GCCATGTGAGCACCGGCGGCGGCGTGAGCCTGGAACTGCTGGAAGGCAAAGTGC |
| A397V_R | GCACTTTGCCTTCCAGCAGTTCCAGGCTCACGCCGCCGCCGGTGCTCACATGGC |
| S219L_F | CTGATTCAGCTGATTAACAACATGCTGGATAAA |
