# Supplementary material for: Mechanisms of Protein Sequence Divergence and Incompatibility
Source: PLoS Genet. 2013 Jul 25;9(7):e1003665. doi: 10.1371/journal.pgen.1003665 (PMC3723536; doi:10.1371/journal.pgen.1003665)
Supplement: Text S1 — Supplementary information. (DOCX) [file pgen.1003665.s016.docx]

**Supplementary online material**

**Mechanisms of protein sequence divergence and incompatibility**

**Alon Wellner, Maria Raitses Gurevich and Dan S. Tawfik**

*Department of Biological Chemistry, Weizmann Institute of Science, Rehovot 76100, Israel*

**Corresponding author:** Dan S. Tawfik, Ullman building - room 201, *Weizmann Institute of Science, Rehovot 76100,* Israel, 0-972-8-9343637*,*  [dan.tawfik@weizmann.ac.il](mailto:dan.tawfik@weizmann.ac.il),

**Supplementary methods**

**Knockout *E. coli pgk***

Briefly, a PCR product containing a Kanamycin resistance cassette flanked by the upstream and downstream chromosomal regions of *E. coli’*s *pgk* was generated with primers PGK_KO_F and PGK_KO_R and pKD13 plasmid as template. 0.5 μgrams of the PCR product were transformed into electrocompetent *E. coli* strain MG1655 cells, harboring a plasmid with a temperature sensitive origin of replication and encoding the λ-red recombinase (pKD46). The cells were plated on glycerol-succinate agar plates (M9 mineral media, 4mM glycerol, 40mM sodium succinate, 0.1% cassamino acids and 25 mg/liter l-tryptophan) with kanamycin. The pKD46 plasmid was lost after incubation at 42^°^C for 5 minutes, resulting in cells with no ampicillin resistance. The chromosomal deletion was verified using PCR and sequencing with primers DEL_VER_F and DEL_VER_R. These primers were also used to sequence the genome of the previously reported *pgk* knockout [1] that was obtained from ATCC (ATCC clone DF576). The obtained Δ*pgk* was unable to grow neither in media containing ≥ 1μM glucose nor in minimal media containing succinate or glycerol alone.

**Cloning, expression and purification of PGK variants**

The pZA vector was constructed in two steps. First, the tetracycline repressor gene was amplified by PCR and cloned to the pZAMCS plasmid (EXPRESSYS; using the Aat2 restriction site) under the constitutive regulation of the ampicillin resistance gene of this plasmid. The multiple cloning site was subsequently modified to add Nco1 and Not1 restriction sites. The nucleotide sequences of human and *M. mazei* *pgk* genes were optimized for *E. coli* codon usage, synthesized (GENESCRIPT, USA) and cloned to the pZA plasmid (plasmid map is available in Figure S12, details and sequence are available as supplementary information below). The K219S mutation in PGK was generated by an all-around PCR with primers K219S_F and K219S_R. The N336S mutation was prepared similarly with primers N336S_F and N336S_R. The S219K mutation in *M. mazei* PGK was incorporated using primers MTM_K219S_F and MTM_K219S_R. Mutation E403D was incorporated using E403D_F and E403D_R. Mutation M239I with M239I_F and M239I_R. Mutation A397V with A397V_F and A397V_R. Mutation S219L with S219L_F and S219K_R.

For protein purification, the various *pgk* variants were recloned into plasmid pPAL8 (BIORAD) using Nco1 and Not1 sites. The plasmids were transformed into *E. coli* BL-21 cells. Transformed cells were grown in 2YT media to mid-log phase, induced by IPTG, and incubated with shaking over night at 25^°^C. Cells were resuspended in a 50mM TRIS-HCl pH 7.5 buffer with 2mM DTT, 1 unit DNase1 (New England Biolabs) and protease inhibitor cocktail (P8849 SIGMA) and lysed by sonication. Purification was performed with the profinity eXACT column (BIORAD) applying standard protocol, and the eluted enzyme variants were dialyzed three times against the resuspension buffer. SDS-PAGE indicated ≥90% purity. Further purification by ion exchange did not enhance the enzyme’s specific activity.

**Ion exchange**

After elution from the profinity eXACT column the proteins were dialyzed against 50mM MES pH 6 buffer with 2mM DTT (buffer A) and loaded onto a strong cation exchange column (HiTrap SP FF, GE) using HPLC with a constant flow rate of 2ml per min. The column was then washed with approximately 20 column volumes of buffer A and was monitored by a UV detector until a baseline was reached. The protein was eluted with a linear NaCl gradient of 0 to 0.5 M in buffer A. 20 fractions of 2ml were collected. UV absorption and SDS-PAGE identified fractions 9-11 as containing PGK. These fractions were pooled and dialyzed against 50mM TRIS-HCl pH 7.5 buffer with 2mM DTT and concentrated using an Amicon Ultra concentrator (Ultracel 10,000 MWCO, Millipore).

**Activity measurement in cell lysates**

The Δ*pgk* strain harboring the pZA human-PGK plasmid and the parental *E. coli K12* strain MG1655 were grown in glycerol-succinate media supplemented with 5mM glucose and various AHT concentrations to OD_600_ of 0.5. Aliquots of cultures (1ml) were centrifuged; the pellets were resuspended in 200μl resuspension buffer (see above) and lysed by sonication in a rotating water bath device until complete clearance was observed. The lysates were centrifuged for 30 min at *14,000* and 20μl of the clarified supernatants were used to determine a PGK activity. The reaction conditions were similar to the ones stated below.

**Gene shuffling**

The *pgk* gene library of the first round was amplified by PCR with primers pZ_F and pZ_R. The purified PCR product (7 μg) was digested with 0.005U DNase1 (NEB) at 37^°^C for 2 min. The reaction was terminated with 10μl of 0.5 M EDTA, and heating to 90^°^C for 15 min, and was run on a 2% agarose gel. Fragments of 50-150 bp length were excised and purified from the gel. The purified fragments (100ng) were reassembled using PCR with the following thermocycling conditions: one denaturation step at 96^°^C for 2.5 min, 35 cycles composed of: (i) a denaturation step at 94^°^C (40 s); (ii) ten successive hybridization steps separated by 3^°^C each, from 66^°^C to 43^°^C, for 1.5 min each (total 13.5 min), and (iii) an elongation step of 1.66 min at 72^°^C. Finally, a 10 min elongation step at 72^°^C was performed. The assembly product was amplified by a nested PCR reaction with primers NSTD_F and NSTD_R, digested with Nco1 and Not1 (Fermentas) and cloned to the pZE plasmid.

**Prevention of mutations at position 219**

Upon preparation of each library, two PCR reactions were initially conducted. The PCRs were performed using one primer that anneals to the plasmid, flanking the *pgk* gene, and a reverse primer that anneals to the *pgk* gene with its 3’ end at position 219 (Figure S11). The primers were designed to amplify position 219 with a serine codon and contain a Bpm1 recognition sequence 5’ to the 219 codon. Bpm1 is a restriction endonuclease that digests at a fixed interval from its recognition sequence, both at the sense and at anti-sense strands, thus creating sticky ends at position 219. This strategy prevents having lysine at position 219 but does not eliminate mutations in the surroundings of this position. After digestion and DNA purification, both PCR products were ligated, amplified again by PCR with primers pZ_F and pZ_R, digested with Nco1 and Not1 and cloned for selection.

**PGK enzymatic assay**

The catalytic efficiency (*k_cat_/K_M_*) of PGK is typically measured for the reverse reaction (3-phosphoglycerate + ATP 🠢 1,3-bisphosphoglycerate + ADP). However, by the Haldane relationship, the same *k_cat_/K_M_* should hold for the forward reaction. Thus, PGK variants were assayed by monitoring the decline of OD_340_ in a coupled assay with GAPDH and different concentrations of ATP. GAPDH from *E. coli* was overexpressed in an AG1 *E. coli* strain containing the pCA24N-*gap* plasmid [2], and purified on a nickel-NTA column. The reactions were performed in 20mM TRIS-HCl buffer pH 7.5, 10mM DTT, 5mM 3-phosphoglyceric acid, 5mM MgCl_2_, 0.2mM NADH and 0.05 mg/ml GAPDH*.* The Michaelis-Menten plots of PGK show complex behavior [3] that differed from one variant to another, thus *k_cat_/K_M_* values were calculated by fitting the initial velocities at ATP concentrations of up to 0.05 mM to a linear regression function.

When substrate concentrations ([S]_0_)are much smaller than the *K_M_*, the Michaelis-Menten equation:

υ_0_ = υ_max_ [S]_0_/(*K_M_*+[S]_0_)

; takes the pseudo-first order form of:

υ_0_ = [S]_0_ (υ_max_ /*K_M_*)

When plotting initial velocity against substrate concentrations (at [S]_0_ ≤ 0.05 mM), the slope of the linear curve corresponds to υ_max_ /*K_M_.* Indeed, all our data fit linear regression with R≥ 0.98.

The kinetic parameters of *M. mazei* PGK and its S219K mutant could not be determined since under the conditions that were used with human and *E. coli* PGKs, only very weak activity was observed.

**Diversification of position 219**

The two libraries were constructed by using a pZA plasmid harboring either WT PGK or variant G4-v4 as templates for allaround PCR. The PCRs were conducted similarly to the preparation of the lysine variants but by using primers NNS_F and S219K_R. The libraries were transformed into Δ*pgk* cells and plated on selection plates with 20 ng/ml AHT. A fraction of the transformation was plated on glycerol-succinate plates containing ampicillin to measure transformation efficiency. The clones were sequenced from the selection plates of each library. The plasmids from each of the seven viable variants of the G4-v4 library were extracted and re-transformed to Δ*pgk* cells. After re-validation of the sequences, their growth rate was measured. They are shown in supplementary Figure 6.

**Details regarding the pZA plasmid**

Nucleotide sequence of the plasmid:

ctcgagtccctatcagtgatagagattgacatccctatcagtgatagagatactgagcacatcagcaggacgcactgaccgaattcattaaagaggagaaaggtaccgggccccccctcgaggtcgacggtatcgataagctaaccatgggcctgagcaacaaactgaccctggataaactggatgtgaaaggcaaacgcgtggtgatgcgcgtggattttaacgtgccgatgaaaaacaaccagattaccaacaaccagcgcattaaagcggcggtgccgagcattaaattttgcctggataacggcgcgaaaagcgtggtgctgatgagccatctgggccgcccggatggcgtgccgatgccggataaatatagcctggaaccggtggcggtggaactgaaaagcctgctgggcaaagatgtgctgtttctgaaagattgcgtgggcccggaagtggaaaaagcgtgcgcgaacccggcggcgggcagcgtgattctgctggaaaacctgcgctttcatgtggaagaagaaggcaaaggcaaagatgcgagcggcaacaaagtgaaagcggaaccggcgaaaattgaagcgtttcgcgcgagcctgagcaaactgggcgatgtgtatgtgaacgatgcgtttggcaccgcgcatcgcgcgcatagcagcatggtgggcgtgaacctgccgcagaaagcgggcggctttctgatgaaaaaagagctcaactattttgcgaaagcgctggaaagcccggaacgcccgtttctggcgattctgggcggcgcgaaagtggcggataaaattcagctgattaacaacatgctggataaagtgaacgaaatgattattggcggcggcatggcgtttacctttctgaaagtgctgaacaacatggaaattggcaccagcctgtttgatgaagaaggcgcgaaaattgtgaaagatctgatgagcaaagcggaaaaaaacggcgtgaaaattaccctgccggtggattttgtgaccgcggataaatttgatgaaaacgcgaaaaccggccaggcgaccgtggcgagcggcattccggcgggctggatgggcctggattgcggcccggaaagcagcaaaaaatatgcggaagcggtgacccgcgcgaaacagattgtgtggaacggcccggtgggcgtgtttgaatgggaagcgtttgcgcgcggcaccaaagcgctgatggatgaagtggtgaaagcgaccagccgcggctgcattaccattattggcggcggcgataccgcgacctgctgcgcgaaatggaacaccgaagataaagtgagccatgtgagcaccggcggcggcgcgagcctggaactgctggaaggcaaagtgctgccgggcgtggatgcgctgagcaacatttaagcggccgcaaacatggtacgcgtgctagaggcatcaaataaaacgaaaggctcagtcgaaagactgggcctttcgttttatctgttgtttgtcggtgaacgctctcctgagtaggacaaatccgccgccctagacctagggatatattccgcttcctcgctcactgactcgctacgctcggtcgttcgactgcggcgagcggaaatggcttacgaacggggcggagatttcctggaagatgccaggaagatacttaacagggaagtgagagggccgcggcaaagccgtttttccataggctccgcccccctgacaagcatcacgaaatctgacgctcaaatcagtggtggcgaaacccgacaggactataaagataccaggcgtttccccctggcggctccctcgtgcgctctcctgttcctgcctttcggtttaccggtgtcattccgctgttatggccgcgtttgtctcattccacgcctgacactcagttccgggtaggcagttcgctccaagctggactgtatgcacgaaccccccgttcagtccgaccgctgcgccttatccggtaactatcgtcttgagtccaacccggaaagacatgcaaaagcaccactggcagcagccactggtaattgatttagaggagttagtcttgaagtcatgcgccggttaaggctaaactgaaaggacaagttttggtgactgcgctcctccaagccagttacctcggttcaaagagttggtagctcagagaaccttcgaaaaaccgccctgcaaggcggttttttcgttttcagagcaagagattacgcgcagaccaaaacgatctcaagaagatcatcttattaatcagataaaatattactagatttcagtgcaatttatctcttcaaatgtagcacctgaagtcagccccatacgatataagttgttactagtgcttggattctcaccaataaaaaacgcccggcggcaaccgagcgttctgaacaaatccagatggagttctgaggtcattactggatctatcaacaggagtccaagcgagaacttcaggtggcacttttcggggaaatgtgcgcggaacccctatttgtttatttttctaaatacattcaaatatgtatccgctcatgagacaataaccctgataaatgcttcaataatattgaaaaaggaagagtatgagtattcaacatttccgtgtcgcccttattcccttttttgcggcattttgccttcctgtttttgctcacccagaaacgctggtgaaagtaaaagatgctgaagatcagttgggtgcacgagtgggttacatcgaactggatctcaacagcggtaagatccttgagagttttcgccccgaagaacgttttccaatgatgagcacttttaaagttctgctatgtggcgcggtattatcccgtattgacgccgggcaagagcaactcggtcgccgcatacactattctcagaatgacttggttgagtactcaccagtcacagaaaagcatcttacggatggcatgacagtaagagaattatgcagtgctgccataaccatgagtgataacactgcggccaacttacttctgacaacgatcggaggaccgaaggagctaaccgcttttttgcacaacatgggggatcatgtaactcgccttgatcgttgggaaccggagctgaatgaagccataccaaacgacgagcgtgacaccacgatgcctgtagcaatggcaacaacgttgcgcaaactattaactggcgaactacttactctagcttcccggcaacaattgatagactggatggaggcggataaagttgcaggaccacttctgcgctcggcccttccggctggctggtttattgctgataaatctggagccggtgagcgtggctctcgcggtatcattgcagcactggggccagatggtaagccctcccgtatcgtagttatctacacgacggggagtcaggcaactatggatgaacgaaatagacagatcgctgagataggtgcctcactgattaagcattggtaggaattaatgatgtctcgtttagataaaagtaaagtgattaacagcgcattagagctgcttaatgaggtcggaatcgaaggtttaacaacccgtaaactcgcccagaagctaggtgtagagcagcctacattgtattggcatgtaaaaaataagcgggctttgctcgacgccttagccattgagatgttagataggcaccatactcacttttgccctttagaaggggaaagctggcaagattttttacgtaataacgctaaaagttttagatgtgctttactaagtcatcgcgatggagcaaaagtacatttaggtacacggcctacagaaaaacagtatgaaactctcgaaaatcaattagcctttttatgccaacaaggtttttcactagagaatgcattatatgcactcagcgcagtggggcattttactttaggttgcgtattggaagatcaagagcatcaagtcgctaaagaagaaagggaaacacctactactgatagtatgccgccattattacgacaagctatcgaattatttgatcaccaaggtgcagagccagccttcttattcggccttgaattgatcatatgcggattagaaaaacaacttaaatgtgaaagtgggtcttaaaagcagcataacctttttccgtgatggtaacttcactagtttaaaaggatctaggtgaagatcctttttgataatctcatgaccaaaatcccttaacgtgaggacgtctaagaaaccattattatcatgacattaacctataaaaataggcgtatcacgaggccctttcgtcttcac

Positions of functional regions within the plasmid:

| element | Position |
| --- | --- |
| Tet promoter | 10 - 142 |
| *pgk* gene | 147 - 1400 |
| P15A ori | 1542 - 1334 |
| Ampicillin resistance gene (BLA) | 2587 - 2345 |
| Tetracycline repressor | 3469 - 4078 |

Amino acids sequence of the PGK protein in our construct:

MGLSNKLTLDKLDVKGKRVVMRVDFNVPMKNNQITNNQRIKAAVPSIKFCLDNGAKSVVLMSHLGRPDGVPMPDKYSLEPVAVELKSLLGKDVLFLKDCVGPEVEKACANPAAGSVILLENLRFHVEEEGKGKDASGNKVKAEPAKIEAFRASLSKLGDVYVNDAFGTAHRAHSSMVGVNLPQKAGGFLMKKELNYFAKALESPERPFLAILGGAKVADKIQLINNMLDKVNEMIIGGGMAFTFLKVLNNMEIGTSLFDEEGAKIVKDLMSKAEKNGVKITLPVDFVTADKFDENAKTGQATVASGIPAGWMGLDCGPESSKKYAEAVTRAKQIVWNGPVGVFEWEAFARGTKALMDEVVKATSRGCITIIGGGDTATCCAKWNTEDKVSHVSTGGGASLELLEGKVLPGVDALSNI

A glycine residue was added at position number 2 to create an Nco1 site that included the ATG of the start codon. The positional numbering, however, was kept in accordance to human PGK.

Complete multiple sequence alignment and tree files can be distributed upon request: Wellner.alon@gmail.com

**Additional references**

1. Irani MH, Maitra PK (1977) Properties of Escherichia coli mutants deficient in enzymes of glycolysis. J Bacteriol 132: 398-410.

2. Kitagawa M, Ara T, Arifuzzaman M, Ioka-Nakamichi T, Inamoto E, et al. (2005) Complete set of ORF clones of Escherichia coli ASKA library (a complete set of E. coli K-12 ORF archive): unique resources for biological research. DNA Res 12: 291-299.

3. Flachner B, Varga A, Szabo J, Barna L, Hajdu I, et al. (2005) Substrate-assisted movement of the catalytic Lys 215 during domain closure: site-directed mutagenesis studies of human 3-phosphoglycerate kinase. Biochemistry 44: 16853-16865.

4. Szilagyi AN, Vas M (1998) Anion activation of 3-phosphoglycerate kinase requires domain closure. Biochemistry 37: 13910.

5. Szabo J, Varga A, Flachner B, Konarev PV, Svergun DI, et al. (2008) Communication between the nucleotide site and the main molecular hinge of 3-phosphoglycerate kinase. Biochemistry 47: 6735-6744.

6. Sambrook J (2001) Molecular Cloning: A laboratory manual 3rd eddition. Cold Spring Harbor Laboratory Press.

7. Lee TS, Krupa RA, Zhang F, Hajimorad M, Holtz WJ, et al. (2011) BglBrick vectors and datasheets: A synthetic biology platform for gene expression. J Biol Eng 5: 12.
